# Highly specific host-pathogen interactions influence *Metarhizium brunneum* blastospore virulence against *Culex quinquefasciatus* larvae

**DOI:** 10.1080/21505594.2018.1509665

**Published:** 2018-09-12

**Authors:** Abeer M. Alkhaibari, Alex M. Lord, Thierry Maffeis, James C. Bull, Fabio L. Olivares, Richard I. Samuels, Tariq M. Butt

**Affiliations:** a Department of Biosciences, College of Science, Swansea University, Swansea, United Kingdom; b Department of Biology, Faculty of Science, Tabuk University, Tabuk, Kingdom of Saudi Arabia; c Centre for Nanohealth, College of Engineering, Swansea University, Swansea, United Kingdom; d Department of Cell and Tissue Biology, State University of North Fluminense Darcy Ribeiro, Campos dos Goytacazes, Brazil; e Department of Entomology and Plant Pathology, State University of North Fluminense Darcy Ribeiro, Campos dos Goytacazes, Brazil

**Keywords:** Mosquito, blastospores, conidia, adhesion, immune responses, infection processes, atomic force microscopy, cuticle

## Abstract

Entomopathogenic fungi are potential biological control agents of mosquitoes. Our group observed that not all mosquitoes were equally susceptible to fungal infection and observed significant differences in virulence of different spore types. Conidiospores and blastospores were tested against *Culex quinquefasciatus* larvae. Blastospores are normally considered more virulent than conidia as they form germ tubes and penetrate the host integument more rapidly than conidia. However, when tested against *Cx. quinquefasciatus*, blastospores were less virulent than conidia. This host-fungus interaction was studied by optical, electron and atomic force microscopy (AFM). Furthermore, host immune responses and specific gene expression were investigated. *Metarhizium brunneum* (formerly *M. anisopliae*) ARSEF 4556 blastospores did not readily adhere to *Culex* larval integument and the main route of infection was through the gut. Adhesion forces between blastospores and *Culex* cuticle were significantly lower than for other insects. Larvae challenged with blastospores showed enhanced immune responses, with increased levels of phenoloxidase, glutathione-S-transferase, esterase, superoxide dismutase and lipid peroxidase activity. Interestingly, *M. brunneum* pathogenicity/stress-related genes were all down-regulated in blastospores exposed to *Culex*. Conversely, when conidia were exposed to *Culex*, the pathogenicity genes involved in adhesion or cuticle degradation were up-regulated. Delayed host mortality following blastospore infection of *Culex* was probably due to lower adhesion rates of blastospores to the cuticle and enhanced host immune responses deployed to counter infection. The results here show that subtle differences in host-pathogen interactions can be responsible for significant changes in virulence when comparing mosquito species, having important consequences for biological control strategies and the understanding of pathogenicity processes.

## Introduction

Entomopathogenic fungi (EPF) of the order Hypocreales are major components of integrated pest management (IPM) programs and they have the potential to play an important role in the biological control of mosquitoes that transmit human and animal diseases [1]. Different fungal isolates can exhibit considerable variation in their virulence levels and host ranges [2,3]. Some *Metarhizium* species (e.g. *Metarhizium brunneum, Metarhizium robertsii* and *Metarhizium anisopliae*) have a wide range of insect hosts, whereas others (e.g. *Metarhizium acridum, Metarhizium album*) are more specific for certain insects such as orthopterans or hemipterans [4,5]. Recently, significant insights into the molecular mechanisms controlling host selectivity by EPF have been obtained and specificity-related genes have been characterized. For example, insertion of an esterase gene (*Mest 1*) into a specific locust pathogen, *M. acridum*, enabled it to expand its host range and infect lepidopteran larvae [6].

**Table 1.** 

	Conidial Infections					Blastospore Infections				
Mosquio Species	Pathogenicity/Virulence	Insect Immune response	Fungal pathogenicity gene response	Role of Proteases	Infection mode/Interaction with integument or intestine	Pathogenicity/ Virulence	Insect Immune response	Fungal pathogenicity gene response	Role of Proteases	Infection mode/Interaction with integument or intestine
*Ae. aegypti*	Conidia were less virulent than blastospores [3,12]	No strong defence response. Cecropin downregulation Upregulation of oxidative stress thiol peroxidase [13]	Upregulated of adhesion (*Mad 1* and *Mad2*) and penetration genes (*Pr1, Pr2*) [13]	Important virulence factor [13]	Low levels of adhesion to larval integument. No gut penetration. Accumulation in the gut [13]	More susceptible to blastospores than conidia [3,12]	Upregulated prior to infection [12]	Upregulated prior to invasion [12]	Little or no role in pathogenesis [12]	High levels of cuticle attachment and penetration. Gut penetration [12]
*Cx. quinquefasciatus*	Conidia were more virulent than blastospores [3] and present paper	Significant up- regulation within 12h post-infection (present paper)	Adhesion gene (Mad 1) and penetration genes (Pr1, Pr2) were upregulated. Mad2 was down-regulated (present paper)	Important virulence factor (present paper)	Low levels of adhesion to larval integument. No gut penetration. Accumulation in the gut (unpublished data)	Cx. quinquefasciatus were less susceptible to blastospores than conidia [3] and present paper	Rapidly up-regulated (present paper)	All target genes were downregulated (present paper)	Little or no role in pathogenesis (present paper)	Low levels of adhesion to integument and main infection route is via the gut (present paper)

The infection process and virulence of *M. brunneum* conidia and blastospores against *Ae. aegypti* and *Cx. quinquefasciatus* larvae.

Different species of mosquitoes (as also seen for other insect hosts), exhibit different degrees of susceptibility to different fungal strains, formulations and propagule types [3,7–11]. Elucidation of the underlying pathogenicity mechanisms is crucial as this will help identify specificity and virulence determinants, host barriers to infection and ultimately help increase EPF efficacy in the control of insect pest species. For example, Alkhaibari et al. [12]. recently showed that *Aedes aegypti* larvae were significantly more susceptible to blastospores of *M. brunneum* than conidia of the same isolate. The differences in susceptibly of *Aedes* larvae to these two types of fungal propagules were attributed to differences in the infection process. Blastospores readily adhere to the cuticle of *Aedes* larvae, producing copious amounts of mucilage, facilitating rapid cuticle penetration, whilst collateral infection through the intestinal tract contributed to rapid host death. Interestingly, conidia of the same fungus kill *Aedes* larvae using a different process. Conidia do not adhere to the larval cuticle but kill the larvae by protease-induced stress following ingestion of large numbers of these propagules [13,14]. However, the virulence pattern of the two fungal spore forms when tested against *Culex quinquefasciatus* larvae was different to that seen against *Aedes* larvae. Alkhaibari et al. [3] found that *Cx. quinquefasciatus* larvae were less susceptible to *M. brunneum* blastospore infection when compared to conidial infection, whereas the opposite pattern seen for *Ae. aegypti* and *Anopheles stephensi* larvae.

There are many virulence factors that influence fungal pathogenicity in insect hosts [15]. The potency of these virulence determinants is dependent upon pathogen specificity and correct orchestration of virulence genes by a complex signalling apparatus [15]. Adhesins and other adhesion molecules are key pathogenicity determinants since firm adhesion of spores to the host surface is an attribute of virulent fungal strains [16–18]. Normally, the more spores that adhere to the host cuticle, the faster the fungus will kill its host; thus poor adhesion is a feature of hypo-virulent isolates [16,19,20]. Spore attachment is a two-step process. The first step is mediated by preformed physio-chemical properties of the spores themselves e.g. hydrophobic and electrostatic forces [21,22], and the second step involves secretion of enzymes and mucilage. Hydrolytic enzymes degrade the cuticle, release nutrients and facilitate penetration [23–25], while mucilage is often secreted to enhance binding to the host cuticle [26]. The adhesin, *Mad1* assists in attachment of fungi to the insect cuticle, thus contributing to pathogenesis. Wang and St Leger [27] found that *Mad1* knockout not only resulted in reduced conidial adhesion to host cuticle, but also reduced germination, blastospore production in the haemolymph and thus a general reduction in virulence.

Differences in susceptibility of mosquito larvae to the two spores forms could also be due to differences in host immune system responses [28–30]. The mosquito immune response involves both cellular and humoral components. The cellular component includes phagocytosis and encapsulation of invading organisms by haemocytes and pericardial cells, while the humoral component includes secretion of inducible antimicrobial peptides [29,31], pattern recognition receptor proteins [32] and activation of the phenoloxidase (PO) cascade, which promotes melanization of the invading parasites and wound healing [33]. The production of oxygen and nitrogen free radicals also occurs in response to infection [34].

Insect cells are able to protect themselves against fungal infection through the activation of detoxifying systems [15]. Insects utilize reactive oxygen species (ROS) as cytotoxic agents against pathogens, however ROS can cause oxidative stress to both the fungal pathogen and host, leading to DNA and protein damage [35]. The host’s cells can protect themselves against these fluctuations by producing enzymatic and non-enzymatic antioxidants [36,37]. Enhanced activity of esterases has been observed in the fat body and hemolymph of *Leptinotarsa decemlineata* and *Locusta migratoria* infected with *M. anisopliae* [38,39] These enzymes play an important role in host protection against pathogens, where increased activity of these enzymes results in the degradation of toxic molecules produced during EPF infection [39,40].

In this study, a range of microscopy, biochemical and molecular biology techniques were used to study the interactions between *M. brunneum* blastospores and *Cx. quinquefasciatus* larvae during the infection process to gain a better understanding of why this type of propagule was less virulent against this host than that observed against *Ae. aegypti*. The study provides novel perspectives on the mechanisms of fungal infection, the evolution of insect-fungus interactions and describes the role of the immune system as a vital defence against EPF.

## Results

### Differences in susceptibility of Cx. *quinquefasciatus* larvae to *M. Brunneum* blastospores and conidia

**Figure 1.**
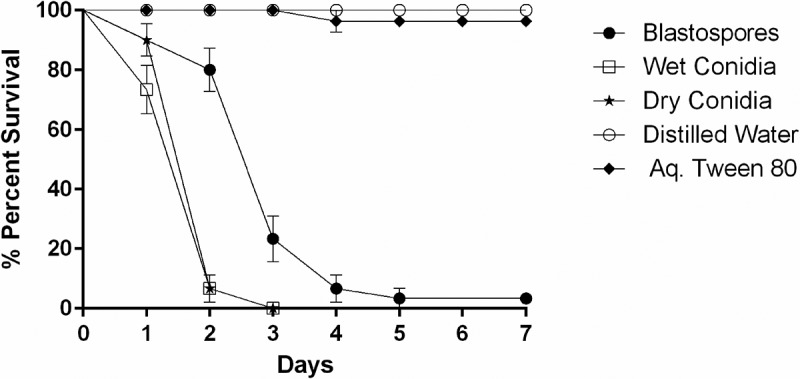
Survival dynamics of 3^rd^-4^th^ instar *Culex quinquefasciatus* larvae (n = 30, 10 larvae per replicate) exposed for 7 days to water treated with *M. brunneum* (isolate ARSEF 4556). Conidia were applied either as dry powder or as a suspension in 0.03% aq. Tween 80, while blastospores were suspended in distilled water. The control treatments were either distilled water or 0.03% aq. Tween 80. Error bars represent ± SE.

The daily survival rates of *Culex* larvae, exposed to three spore formulations, were significantly reduced when compared with control survival rates (X^2^ = 175.703, df = 4, *P < *0.001, Figure 1). When comparing larval mortality using different spore formulations, there was a significant difference between blastospores and conidia whether dry (X^2^ = 32.539, df = 4, *P < *0.001) or wet (X^2^ = 34.096, df = 4, *P < *0.001). The median lethal time (LT_50_) was significantly lower for both dry (LT_50_ = 1.45 days, 95% ci: 1.26–1.64) and wet conidia (LT_50_ = 1.27 days, 95% ci: 1.08–1.47) when compared to blastospores (LT_50_ = 2.68 days, 95% ci: 2.39–2.97). However, there was no significant differences in survival rates when comparing larvae treated with either wet or dry conidia (X^2^ = 1.641, df = 4, *P *= 0.200).

### Protease inhibitor influences virulence of conidia but not blastospores

**Figure 2.**
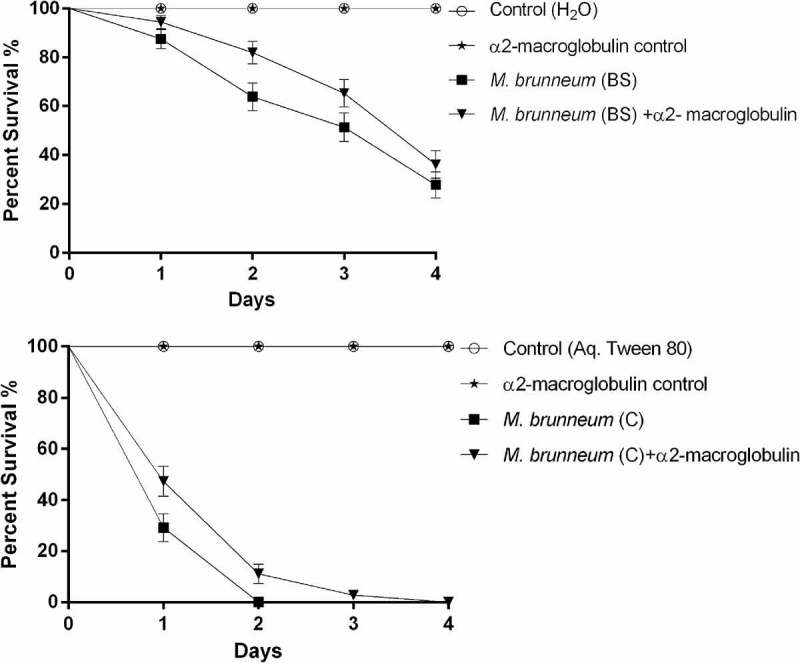
Survival of *Culex quinquefasciatus* larvae exposed to blastospores (BS) and conidia (C) of *M. brunneum* in the presence and absence of a protease inhibitor. *Cx. quinquefasciatus* (n = 72, 24 larvae per replicate) were exposed to *M. brunneum* blastospores and conidia (10^7^ spores ml^−1^) with and without the protease inhibitor α2-macroglobulin. Controls consisted of either distilled water or 0.03% aq. Tween 80 with and without the inhibitor. Error bars represent **± **SE.

No statistically significant difference in virulence was observed when *Cx. quinquefasciatus* larvae were exposed to blastospores with or without the protease inhibitor α2-macroglobulin (X^2^ = 2.833, df = 2, *P *= 0.092, Figure 2). However, the survival of *Culex* larvae exposed to conidia was significantly lower in the absence of the protease inhibitor (X^2^ = 8.949, df = 2, *P *= 0.003, Figure 2).

### Blastospore infection process

**Figure 3.**
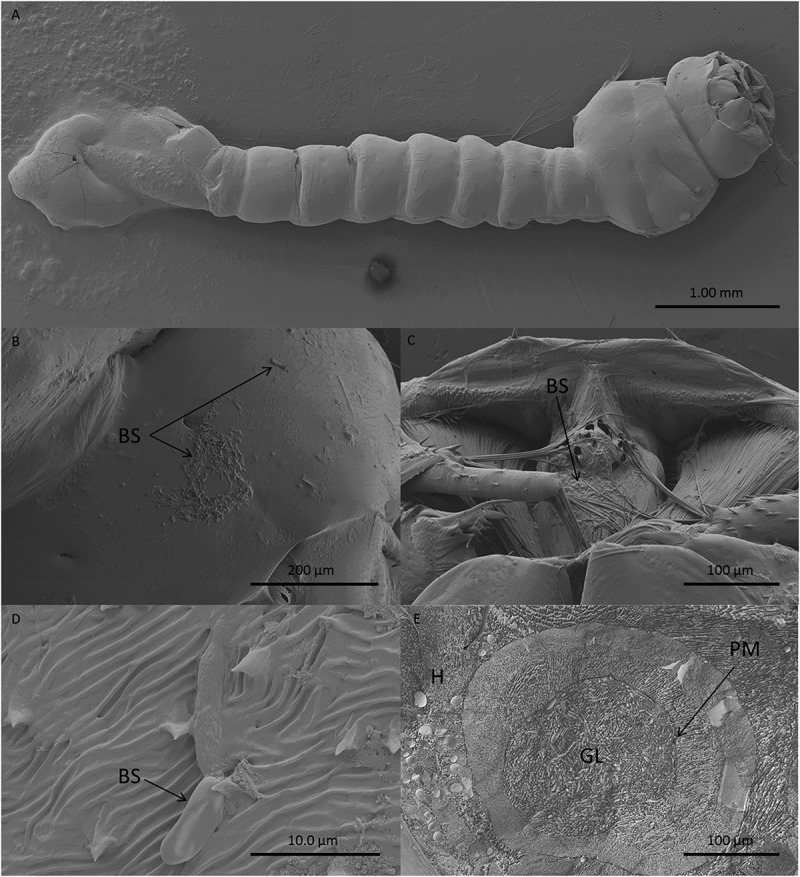
Scanning electron microscopy of *Culex quinquefasciatus* larvae infected with blastospores (BS) of *Metarhizium brunneum* ARSEF 4556. Larvae exposed to 10^7^ blastospores ml^−1^, 48 hr post-inoculation, were examined using Cryo-SEM. (a). Distribution of blastospores on larval cuticle. (b) Some blastospores were found attached to the surface of the head and (c) around mosquito mouthparts. (d) Few blastospores were observed attached to abdominal segments. (e) Cross section of infected larva showing that blastospores of *M. brunneum* had been ingested by the larvae and occluded the gut lumen (GL). PM: peritrophic membrane, H: haemocoel.

Light and electron microscopy studies were undertaken to determine the mode of action of *M. brunneum* (ARSEF 4556) blastospores when infecting *Cx quinquefasciatus* larvae. In general, cryo-scanning election microscopy (cryo-SEM) showed little attachment of blastospores to the mosquito larval integument (Figure 3(a)). However, blastospore adhesion was observed at higher densities on the siphon and head regions, in particular around the mouthparts (Figure 3(b,c)). Few blastospores were observed on the abdominal segments of *Culex* larvae, (Figure 3(a–d)).

**Figure 4.**
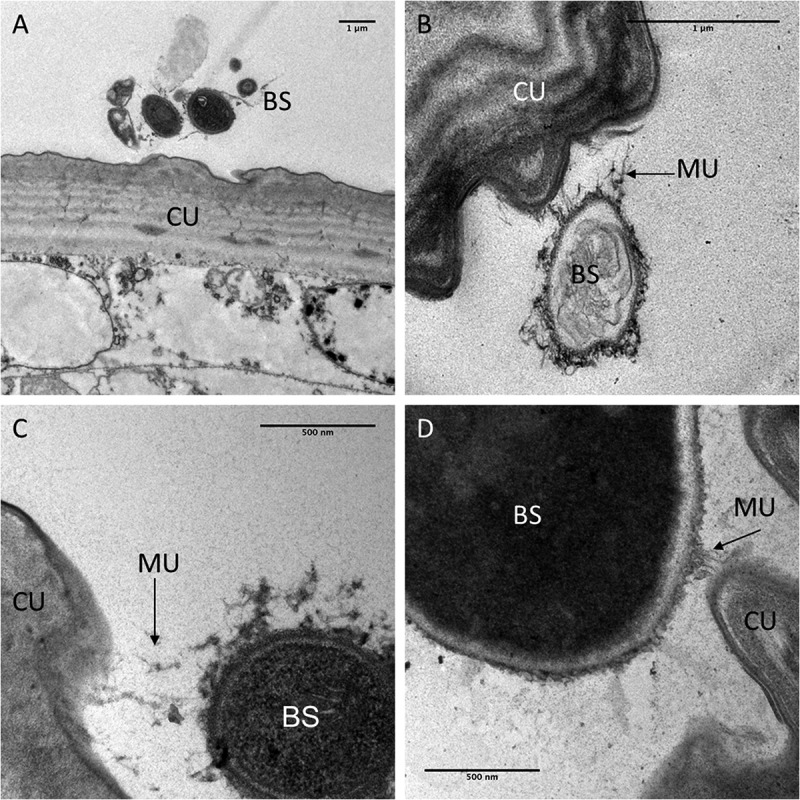
Transmission electron microscopy of interactions between the integument and *Metarhizium brunneum* ARSEF 4556 blastopsores in the abdominal region of *Culex quinquefasciatus* larvae 48 hr post-inoculation. (a). Low magnification section showing grouping of blastospores (BS) near the abdominal cuticle (CU). (b). Higher magnification showing a blastospore secreting mucilage (MU) strands in the direction of the host cuticle surface. (c & d). host-pathogen interactions with mucilage forming loose connections with the cuticle surface.

TEM observations carried out to observe the interaction between blastospores and the larval cuticle showed no attachment to the abdomen (Figure 4(a–d)). Although mucilage was produced by the blastospores, this did not appear to facilitate attachment to the cuticle surface. Some mucilage strands managed to make contact with the cuticle surface (Figure 4(d)) but no blastospores were observed in close contact with the cuticle.

**Figure 5.**
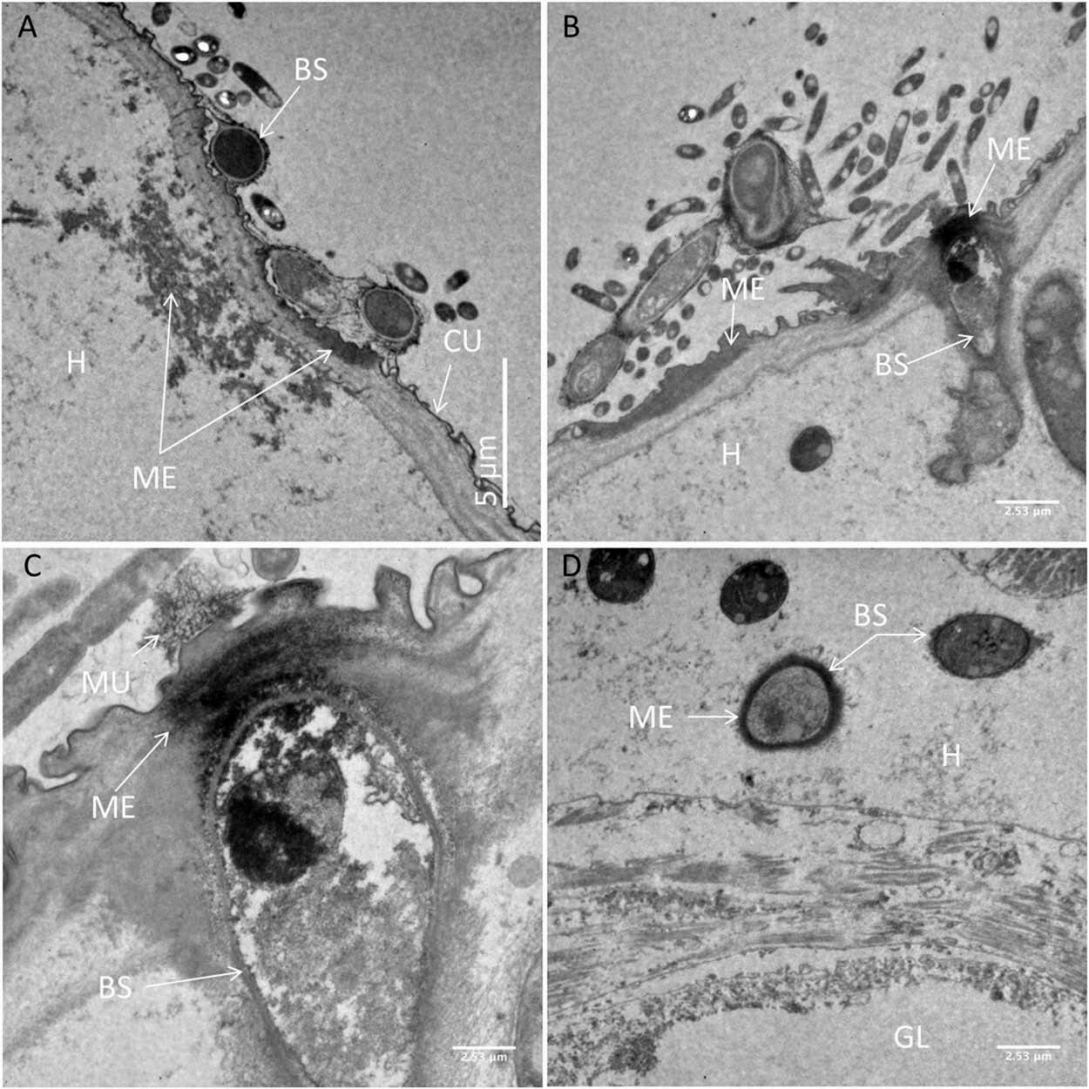
Transmission electron microscopy of *Culex quinquefasciatus* larval head/thorax region showing defence responses to *Metarhizium brunneum* ARSEF 4556 blastospore infection, 48 hr post-inoculation. (a). Humoral and cuticular melanisation (ME). Melanization in the haemolymph takes place close to or around circulating blastospores (BS) and beneath integument at sites where blastospores are adhering or attempting to penetrate the cuticle (CU). The cuticle is also melanized at adhesion or putative infection sites. (b). Blastospore penetrating the cuticle and melanisation response to invasion. (c). Increased magnification showing the melanisation response and the possible stress effect on the blastospore integrity (cytoplasm retraction). (d). Intestinal tract showing blastospores near the basal region of the gut epidermis. Gut lumen (GL), cuticle (CU), mucilage (MU), haemocoel (H).

In the head region of *Culex* larvae infected with blastospores, deposition of melanin was observed in the haemolymph and in the cuticle (Figure 5(a)). The melanisation appeared to be humoral as opposed to cellular, since haemocytes were not evident in both thick and thin sections examined by LM and TEM, respectively. Melanisation of the cuticle often extended over the length of the blastospore that had made contact with the cuticle (Figure 5(a,b)). The contour of the fungus usually complemented that of the host cuticle revealing intimate contact before detachment during sample preparation (Figure 5(a,b)). The electron opaque extracellular matrix that acted as a bridge between the fungus and cuticle also detached with only traces of the material remaining attached to the cuticle (Figure 5). Melanisation was often limited to the outer layers of the cuticle but was particularly intense at penetration sites (Figure 5(a–c)). Amorphous zones of diffuse granular melanin were observed in the haemolymph. Whereas some of the melanin was observed close to penetration sites, similar material was also present close to or surrounding the blastospores (Figure 5(c–d)). Not all circulating blastospores were melanised but some were clearly more melanised than others, being enveloped in a relatively thick, electron opaque capsule (Figure 5(d)). Melanisation was not extensive since it was not observed in all individuals examined nor was it readily evident in thick sections observed using light microscopy (Figure 8). LM confirmed TEM observations that haemocytes did not appear to play a significant role in defence; no cells were observed phagocytosing or encapsulating blastospores and they were also absent or inconspicuous at sites of melanisation.

**Figure 6.**
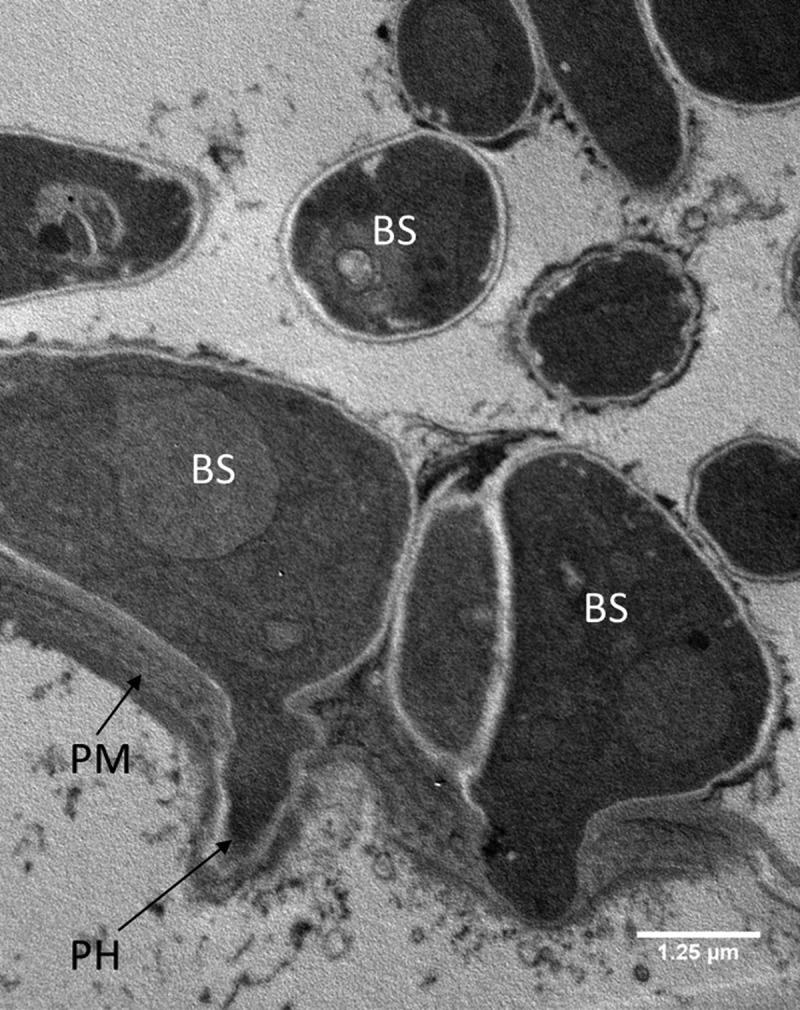
Transmission electron microscopy of *Metarhizium brunneum* ARSEF 4556 blastospores in gut lumen penetrating gut wall of *Culex quinquefasciatus* larva, 48 hr post-inoculation. Blastospores (BS), Gut lumen (GL), Peritrophic membrane (PM), Penetration hypha (PH).

**Figure 7.**
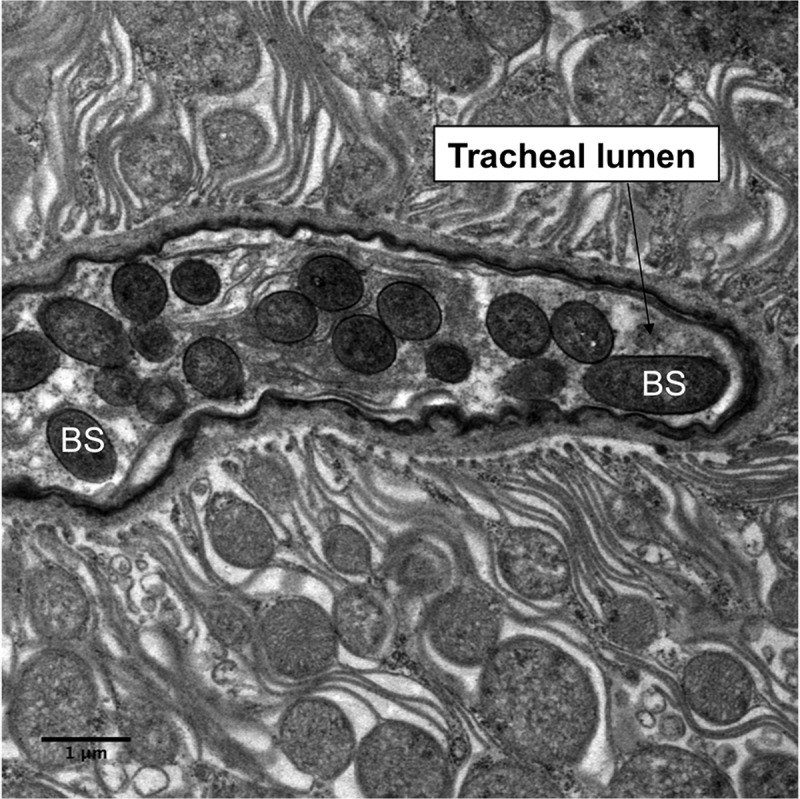
Transmission electron microscopy of *Metarhizium brunneum* ARSEF 4556 blastospores (BS) in tracheal lumen of *Culex quinquefasciatus* larva, at 48 hr post-inoculation.

**Figure 8.**
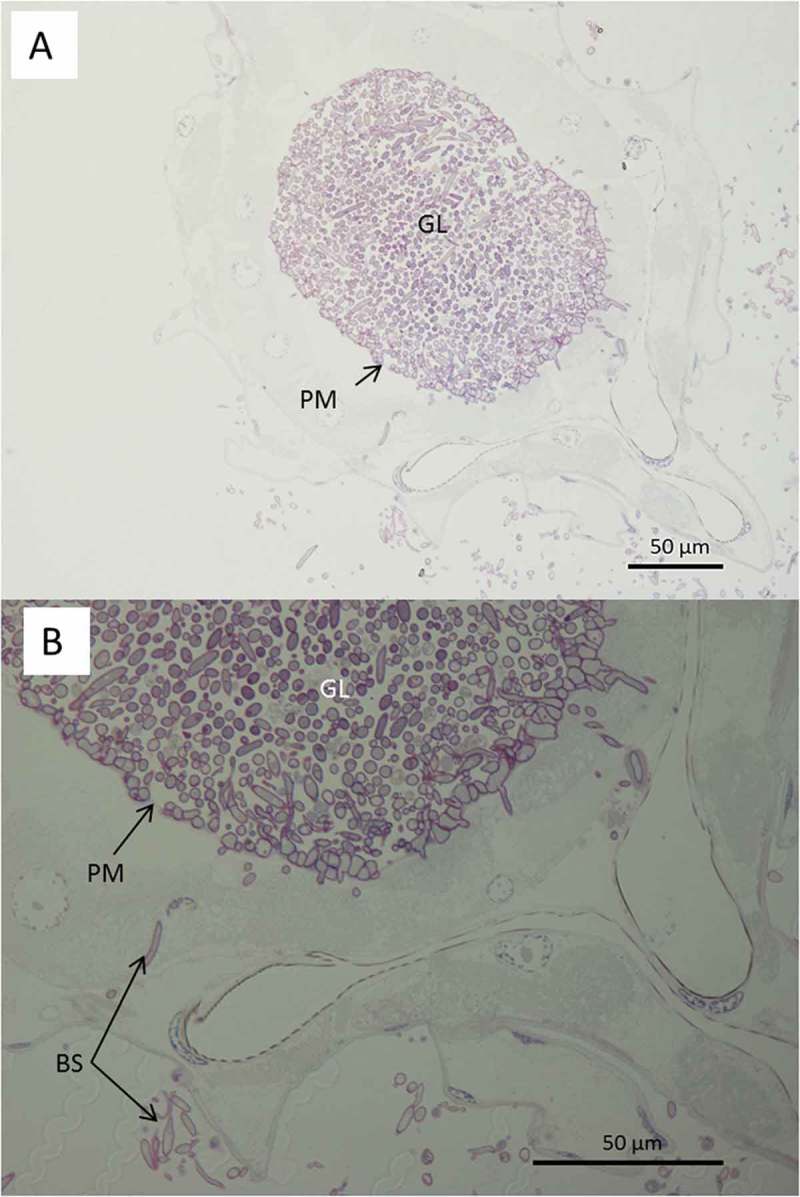
Light microscopy of transverse sections of *Culex quinquefasciatus* larvae 48 hr post infection with *Metarhizium brunneum* ARSEF 4556. (A): Blastospores of *M. brunneum* mostly confined to gut lumen. (B): The blastospores close to the peritrophic membrane are swollen and penetrated the epithelium of midgut to colonize the haemocoel. GL: Gut lumen, PM: peritrophic membrane, BS: Blastospores, H: haemocoel, CU: cuticle surface.

Both light and electron microscopy showed that blastospores were ingested by larvae and concentrated in the gut lumen (Figures 3(e) and 6–8(a, b)). At 48 hr pi it was possible to observe gut epithelial penetration by hyphae (Figure 6). Blastospores that penetrated the gut epithelium and colonized the larval haemocoel have the same appearance as those cultured in liquid media (Figure 8(b)). Blastospores were also observed within the tracheal lumen (Figure 7).

### AFM measurements of blastospore adhesion

**Figure 9.**
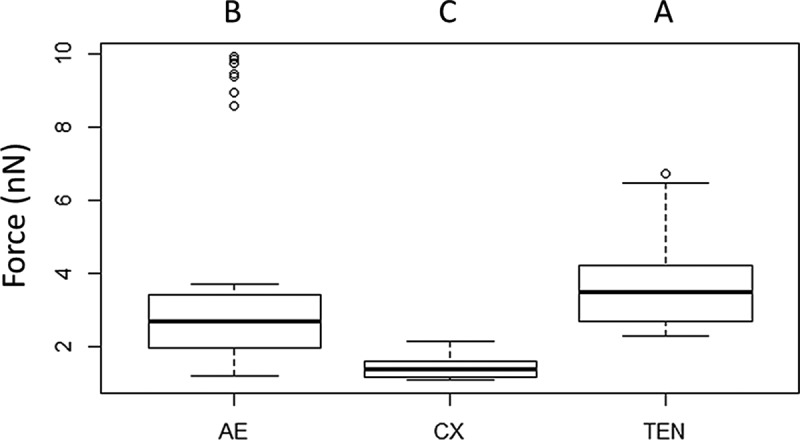
Adhesion forces measured by AFM. The average measured value of adhesion forces between *Metarhizium brunneum* ARSEF 4556 blastospores and *Aedes* (AE), *Culex* (CX) and *Tenebrio* (TEN) cuticles (n = 100). Significant differences were denoted by different letters, Tukey whisker (25–75 percent quartiles). Boxes denote interquartile range, bisected horizontally by median values; whiskers extend to 1.5× interquartile range beyond boxes; outliers are marked as dots beyond whiskers. Different letters (A, B and C) indicate significant differences between species.

**Figure 10.**
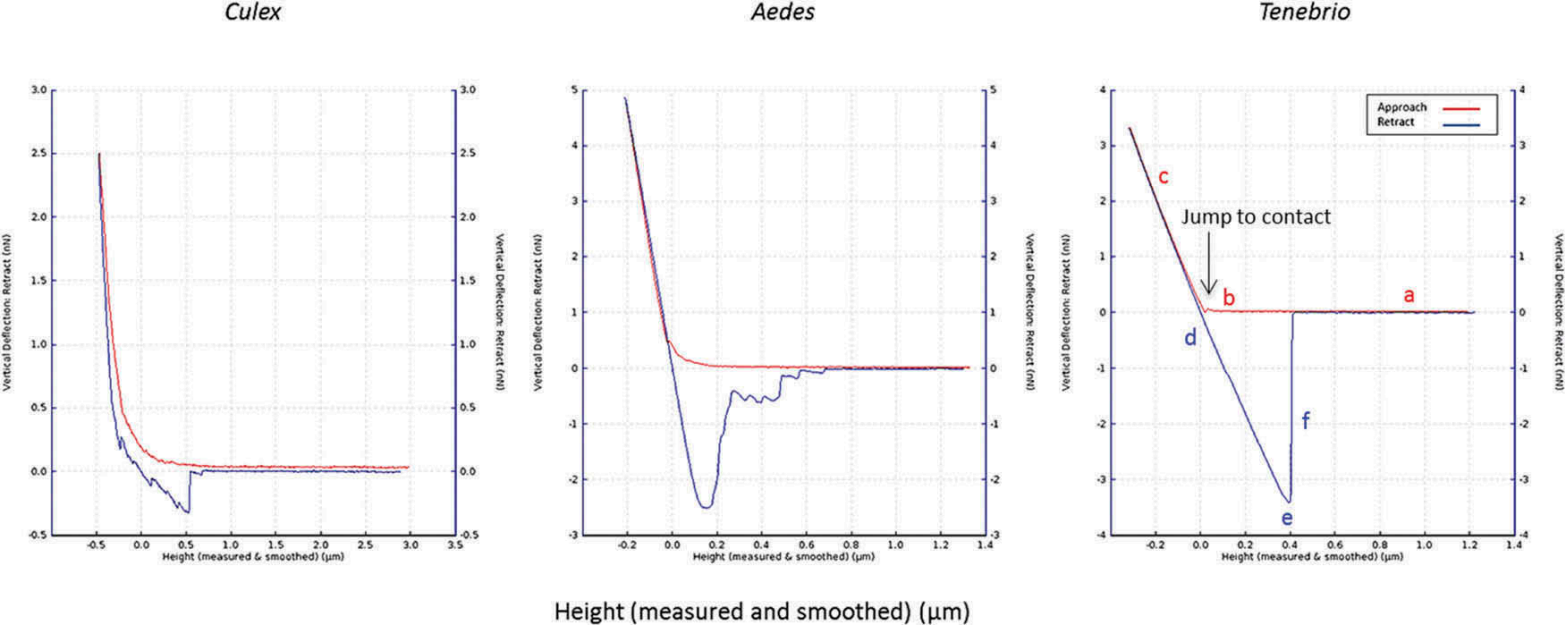
Typical force-distance curves for the interaction of the blastospore probe with larval cuticles of *Culex, Aedes* and *Tenebrio*. The highest adhesion forces were recorded for *Tenebrio* and *Aedes*, while the lowest were recorded for *Culex*. The red and blue lines represent the approach and retraction curves, respectively. Initially, the cantilever is away from the sample surface (a) and there is no interaction. It is then moved to the surface (b) or jump to contact point. The cantilever tip remains in contact as the separation between the cantilever and sample decreases, causing a deflection of the cantilever (c). As the cantilever is retracted the tip remains in contact with surface (d), due to adhesion and the cantilever is deflected downwards (e). Eventually, after the adhesion force has been overcome, the tip breaks free and returns a neutral position (f).

Figures 9 and 10 show results for measurements of the deflection of the AFM-cantilever as a function of the interaction between the “blastospore probe” and cuticle of three different insects. The AFM measurements determined the interaction forces between immobilized blastospores and the abdominal cuticle of terrestrial (*Tenebrio molitor* larvae) and aquatic insects (mosquito larvae).

The adhesion forces measured with the blastospore probe showed significant differences between all three cuticle samples; *Aedes, Culex* and *Tenebrio* (X^2^  = 88.59, *df* = 2, *P* < 0.001, Figure 9). The forces generated between *Tenbrio* larval cuticle and the blastospore probe were significantly higher than for *Aedes* cuticle (Z = −2.57, *P* = 0.005) and *Culex* cuticle (Z = 88.59, *P* < 0.001). A significant difference in the forces was also observed when comparing *Aedes* and *Culex* (Z = 88.59, *P* < 0.001), with the lowest interaction forces seen when testing the blastospore probe against *Culex* larval cuticle in comparison with both *Tenebrio* and *Aedes* larvae.

### Enzymatic stress and immune responses

#### Phenoloxidase (PO)

**Figure 11.**
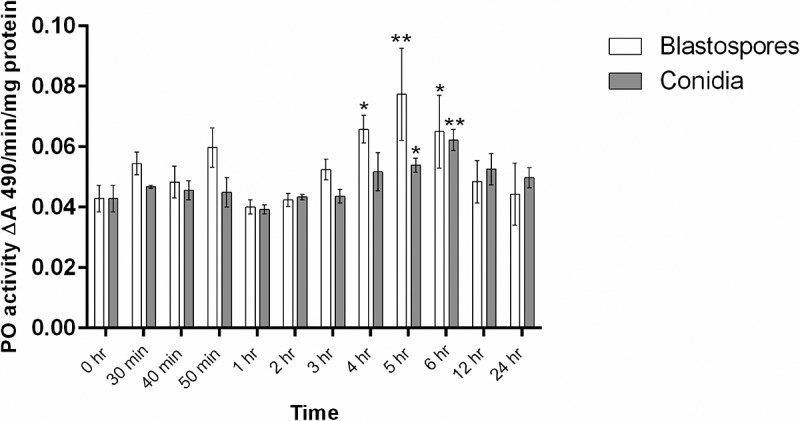
Phenoloxidase (PO) activity in total homogenates of *Cx. quinquefasciatus* larvae (n  =  45, 15 larvae per replicate) at different time points after infection with conidial and blastospore suspensions of *M. brunneum* (10^7^ spores ml^−1^). Data presented as mean activity ± SEM. Significant differences are shown at two levels ** *p *< 0.01, * *p* < 0.05 when compared to PO activity in non-infected control insects (0 hr).

*Culex* larvae infected with blastospores showed significantly higher levels of PO activity 4 hr pi (GLM: Estimate = 0.023, Std. Error = 0.010, t = 2.397, P = 0.025), 5 hr pi (GLM: Estimate = 0.034, Std. Error = 0 .011, t = 3.226, P = 0.004), and 6 hr pi (GLM: Estimate = 0.022, Std. Error = 0.010, t = 2.317, P = 0.030), when compared to control larvae (Figure 11).

PO activity was not higher in the larvae infected with conidia than that seen in uninfected larvae at 4 h. However, the PO levels in the larvae infected with conidia was higher than the control at 5 hr pi (GLM: Estimate = 0.011, Std. Error = 0.005, t = 2.171, P = 0.040) and 6 h pi (GLM: Estimate = 0.019, Std. Error = 0.005, t = 3.793, P = 0.001). At 6 hr pi the level of PO activity in larvae infected with blastospores was higher than that for larvae infected with conidia (GLM: Estimate = −0.024, Std. Error = 0.011, t = -2.046, P = 0.0463). For *Culex* larvae infected with either blastospores or conidia, there were no significant changes in PO activity observed during the early stage of infection (30 min–3 hr).

#### Glutathione-S-transferase (GST) activity

**Figure 12.**
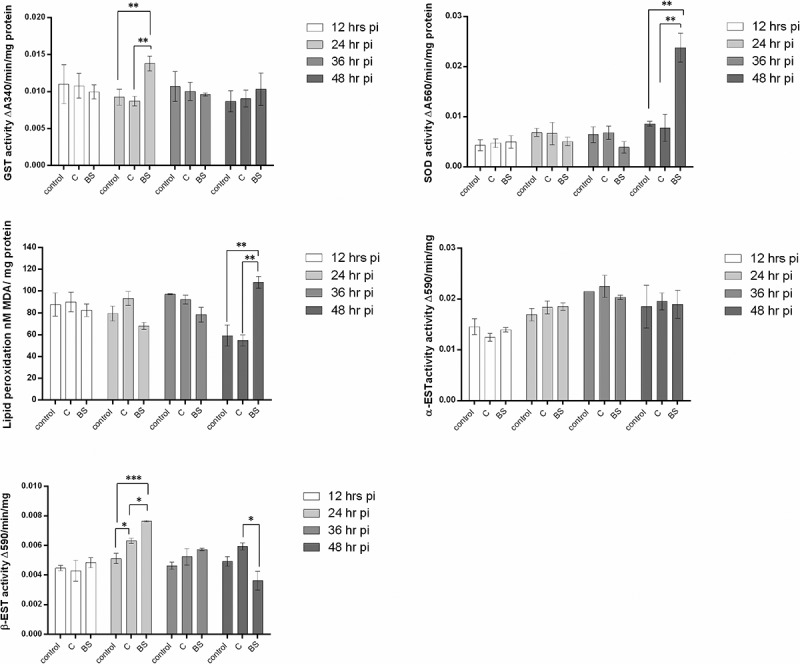
Antioxidant activity in mosquito larvae (n  =  12, 3 larvae per replicate) exposed to *M. brunneum* blastospores (BS) or conidia (C). Activity of glutathione-S-transferase (GST), Superoxide dismutase (SOD), MDA (lipid peroxidase), α-esterase (α-EST) and β-esterase (β-EST) were measured in mosquito larvae exposed to blastospores and conidia. Data presented as means ± SEM. Significant differences are show at three levels ***p < 0.001,**p < 0.01, *p < 0.05. Controls were not exposed to fungal propagules.

The experimental results showed an immune reaction in larvae of *Cx. quinquefasciatus* after infection with *M. brunneum* blastospores when using GST activity as a parameter. GST activity at 24 hr pi was significantly higher in larvae infected with blastospores when compared to larvae infected with conidia (diff = 0.005, [0.001, 0.009], P = 0.020) or control larvae (0.005, [0.0004, 0.009], P = 0.032], with no significant difference between the controls and larvae infected with conidia (0.001 [−0.003, 0.005], P = 0.912, Figure 12)

#### Superoxide dismutase (SOD) activity

On the second day pi with blastospores, the level of SOD activity was significantly higher than for larvae infected with conidia (diff = 0.016, [95% c.i. 0.006, 0.026], P = 0.005) or the controls (diff = 0.015 [0.005, 0.026], P = 0.010). The levels of SOD in controls and larvae infected with conidia were similar (0.001, [−0.009, 0.011], P = 0.966] at the 24 hr pi (Fig .12)

#### Concentration of malondialdehyde (MDA)

On the second day after the *Culex* larvae were inoculated with *M. brunneum*, the concentration of MDA in larvae infected with blastospores was significantly higher than the controls (diff = 48.789, [95% c.i. 18.272, 79.306], P = 0.006] or larvae infected with conidia (53.331, [22.914, 83.987], P = 0.004). Similar MDA activities were observed when comparing controls and larvae infected with conidia (8.642, [−25.876, 35.158], P = 0.889, Figure 12).

#### Esterase (EST) activity

There were no major differences in α-esterase activity between uninfected larvae and *M. brunneum* (blastospores or condia) infected larvae at all time points [(12 h: F_2,6_ = 1.257, P = 0.342); (24 h: F_2,6_ = 0.659, P = 0.551); (36 h: F_2,6_ = 0.698, P = 0.534); 48 h: F_2,9_ = 0.028, P = 0.973); Figure 4]. In contrast, β-esterase activity at 24 hr pi was higher in larvae infected with either conidia or blastospores than the controls (C: diff = −0.001, [95% c.i. lower = −0.002, upper = −0.0002], Tukey’s HSD: P = 0.021; BS: −0.003, [−0.003, −0.002], P < 0.001). The level of β-esterase in larvae infected with *M. brunneum* blastospores was significantly higher than for larvae infected with conidia at 24 hr pi (0.001, [0.0004, 0.002], P = 0.014, Figure 12). However, after 48 hr pi, no significant difference was observed between the controls and larvae infected with blastospores (F_2,9_ = 7.239, P = 0.137) or conidia (F_2,9_ = 7.239, P = 0.272, Figure 12).

### Expression of stress and pathogenicity-related genes in *metarhizium brunneum*

**Figure 13.**
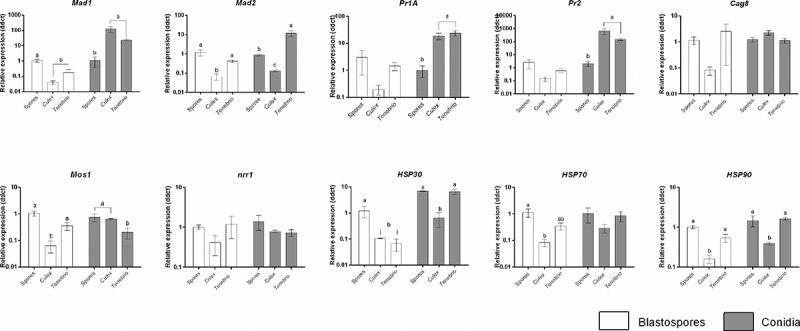
Expression of *Metarhizium brunneum* (AFSEF 4556) virulence and pathogenicity genes (n  =  30, 10 larvae per replicate) . Quantitative PCR was used to analyse expression of pathogenicity related genes in blastospores and conidia of *M. brunneum* at 24 hr. Genes investigated: adhesins (*Mad1, Mad2*), proteases (*Pr1, Pr2*), stress management genes (*HSP30, HSP70* and *HSP90*), an osmosensor (*Mos1*), G-protein signalling regulator (*Cag8*) and nitrogen regulator (*nrr1)*. Data was presented as mean (±SEM) means with different letters at the same time point show statistical differences.

Adhesins (*Mad1, Mad2*), proteases (*Pr1A, Pr2*), regulators of G-protein signalling (*Cag8*), an osmosensor (*Mos1*), nitrogen regulator response (*nrr1*) and the stress management genes (*HSP30, HSP70* and *HSP90*) genes play a fundamental role in fungal virulence and pathogenicity. The expression of these genes was analysed in spores ingested by *Cx. quinquefasciatus* larvae, spore pellets in the absence of *Culex* larvae and infected *T. molitor* (adults) which were used as a positive control (Figure 13).

Expression of *Mad1* in blastospores in infected living *Culex* larvae and *T. molitor* (positive control) was significantly lower than that seen in blastospore pellets in the absence of larvae (diff = −1.414 [95% CI: −2.141, −0.687], Tukey’s HSD: *P* = 0.002; −0.854 [−1.581, −0.127], *P* = 0.026, respectively). A similar expression level of *Mad1* was observed for blastospore infected *Culex* and *T. molitor* (−0.560 [−1.287, 0.167], *P* = 0.122, Figure 13). On the other hand, higher expression levels of *Mad1* were recorded for both *Culex* and *T. molitor* infected with *M. brunneum* conidia than seen in the conidia in the absence of the larvae (2.134 [1.069, 3.198], *P* = 0.002; 1.540 [0.475, 2.604], *P* = 0.010, respectively). The expression level of *Mad1* in *Culex* larvae infected with conidia was similar to that seen in *T. molitor* infected with conidia (0.594 [−0.470, 1.658], *P* = 0.276, Figure 13).

The adhesion gene *Mad2* was significantly down regulated in *Culex* larvae infected with blastospores in comparison with the expression seen in blastospores in absence of *Culex* larvae (−1.229 [−1.837, −0.611], *P* = 0.002) and *Tenebrio* infected with blastospores (−0.831 [−1.450, −0.214], *P* = 0.014). No difference was observed between blastospores in the absence of larvae and *T. molitor* infected with blastospores (0.397 [−0.221, 1.015], *P* = 0.200, Figure 12). While, in conidial applications, the highest expression levels of *Mad2* were detected in *T. molitor* followed by conidia in the absence of *Culex* larvae and *Culex* larvae infected with conidia (Figure 13).

Expression of *Pr1A* at 24 hr pi was similar between all the treatments; blastospores in the absence of *Culex* larvae and *Culex* larvae or *T. molitor* infected with blastospores (F_2,5_ = 1.619, *P* = 0.287, Figure 12). However, significant up-regulation of *Pr1A* in both *Culex* larvae and *T. molitor* infected with conidia were seen in comparison with conidial pellets in the absence of the larvae (1.354 [0.554, 2.154], *P* = 0.006; 1.456 [0.741, 2.172], *P* = 0.003, respectively). The pattern of *Pr1A* expression was similar in infected live *Culex* larvae and *T. molitor* (−0.102 [−0.901, 0.698], P = 0.911, Figure 13).

A similar pattern for *Pr2* was observed in blastospores not exposed to *Culex* larvae, blastospores in infected *Culex* larvae and *T. molitor* at 24 hr pi (F_2,6_ = 1.834, *P* = 0.239). However, the expression levels of *Pr2* were significantly higher in both *Culex* larvae and *T. molitor* infected with conidia than conidia that had not been exposed to *Culex* larvae (2.537 [1.732, 3.343], *P* < 0.001; 1.949 [1.144, 2.755], *P* = 0.001). Both *Culex* larvae and *T. molitor* infected with conidia, had similar expression levels of *Pr2* (0.588 [−0.218, 1.394] *P* = 0.142, Figure 13).

Expression of *Cag8* at 24h pi was similar between all the blastospore treatments; blastospores in absence of larvae, infected *Culex* and *T. molitor* (F_2,6_ = 1.523, *P* = 0.292). This pattern was also observed between all conidial treatments; conidia in the absence of larvae, *Culex* and *T. molitor* infected with conidia (F_2,6_ = 3.098, *P *= 0.119, Figure 13). The expression of *Mos1* in *Culex* larvae infected with blastospores was significantly lower than other treatments; blastospores in the absence of larvae (−1.278 [−2.000, −0.556], *P* = 0.004) and *T. molitor* infected with blastospores (−0.761 [−1.484, −0.038], *P* = 0.041). No statistically significant difference was observed between blastospores not exposed to larvae and *T. molitor* infected with blastospores (0.517 [−0.206, 1.240,], *P* = 0.151, Figure 13). *T. molitor* infected with conidia had the lowest level of *Mos1* among all conidial treatments. Conidia not exposed to larvae and larvae infected with conidia exhibited similar expression level of *Mos1* (0.0235 [−0.558, 0.605], *P* = 0.992, Figure 13). Nitrogen regulator response (*nrr1*) expression had similar levels between all blastospore treatments (F_2,5_ = 2.048, *P* = 0.224). Likewise, all conidial treatments expressed similar levels of *nrr1* (F_2,6_ = 0.837, *P* = 0.478, Figure 13).

Heat shock protein 30 (*HSP30*) expression was significantly lower in both *Culex* and *T. molitor* infected with blastospores when compared to blastospores not exposed *Culex* larvae (−0.975 [−1.922, 0.028], *P* = 0.045; −1.288 [−2.136, 0.441], *P* = 0.010, respectively) and both blastospores in infected *Culex* and *T. molitor* had similar expression levels (−0.314 [−1.261, 0.634), *P* = 0.566). In contrast, *HSP30* expression in conidia was similar for the control (absence of *Culex* larvae) and *T. molitor* treatment (−0.032 [−0.874, 0.811], *P* = 0.992) but respectively lower in the presence of *Culex* (−1.162 [−2.003, −0.319], *P* = 0.015; −1.130 [−1.88, −0.377], *P* = 0.10).

The expression of the heat shock protein 70 (*HSP70*) gene at 24 hr was lowest in *Culex* larvae infected with blastospores, followed by *T. molitor* infected with blastospores and blastospores in absence of the larvae. However, the level of *HSP70* in the three conidial treatments was similar (F_2,6_ = 1.688, *P* = 0.262, Figure 13).

Expression of the heat shock protein 90 (*HSP90*) gene at 24 hr pi was significantly down-regulated in *Culex* infected with blastospores in comparison with blastospores in absence of the larvae (−0.824 [−1.219, −0.430], *P *= 0.002) and *T. molitor* infected with blastospores (−0.548 [−0.989, −0.107], P = 0.022). No difference was observed between blastospores in the absence of the larvae and *T. molitor* infected with blastospores (−0.276 [−0.717, 0.164], *P* = 0.197). Similarly, *Culex* infected with conidia had the lowest expression level of *HSP90*, followed by *T. molitor* infected with conidia and conidia not exposed to larvae. The two conidial treatments; conidia in the absence of the larvae and *T. molitor* infected with conidia had similar expression levels (−0.086 [−0.464, 0.292], *P* = 0.775, Figure 13).

### Expression of antimicrobial and stress-related genes in Culex quiquefasiatus larvae

**Figure 14.**
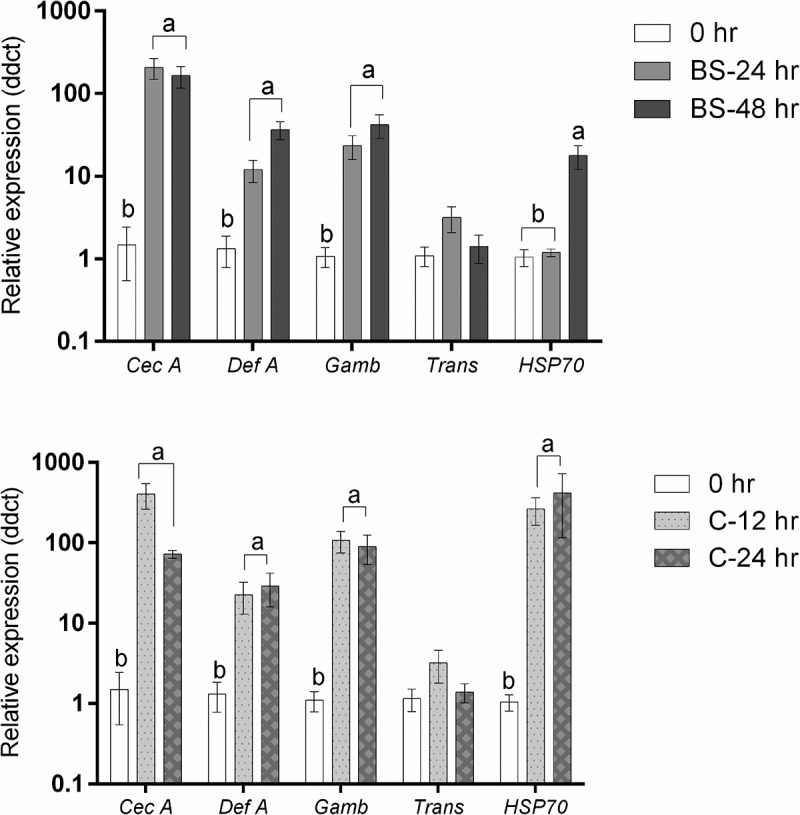
Antimicrobial peptide and stress gene expression in *Culex quinquefasciatus* larvae (n  =  30, 10 larvae per replicate) during infection with *Metarhizium brunneum* blastospores (BS) or conidia (C). Quantitative PCR was used to analyse AMPs and stress gene expression in *Cx. quinquefasciatus* larvae inoculated with *M. brunneum* at 0 hr (uninfected control), 24 hr and 48 hr post-inoculation. *Cec A* (*Cecropin A), Def A* (*Defensin A), Gamb* (*Gambicin), Trans* (*Transferrin*) and *HSP70* (Heat shock protein 70). Data was presented as means (±SEM). Columns with different letters were statistically different.

Figure 14 shows that infection of *Culex* larvae with blastospores of the entomopathogenic fungus *Metarhizium brunneum* had a significant impact on the relative expression of *cecropin A* (F_2,6_ = 48.555, *P* < 0.001), *Defensin A* (F_2,6_ = 19.271, *P* = 0.002), *Gambicin* (F_2,6_ = 30.431, *P* = 0.001) and *HSP70* (F_2,6_ = 28.144, *P* = 0.001), but no effect on expression of *Transferrin* (F_2,6_ = 2.448, *P* = 0.167). *Culex* larvae infected with conidia showed a similar pattern, where there was a significant impact of *M. brunneum* conidia on the relative expression of all studied genes; *Cecropin A* (F_2,6_ = 41.922, *P < *0.001), *Defensin A* (F_2,6_ = 9.040, *P* = 0.015), *Gambicin* (F_2,6_ = 42.124, *P < *0.001), and *HSP70* (F_2,6_ = 43.361, *P < *0.001) except for *Transferrin* which had similar expression levels at all time points examined (F_2,6_ = 1.283, *P* = 0.344).

The expression levels of *Cecropin A, defensin A* and *gambicin* in larvae infected with blastospores were significantly up-regulated at 24 hr pi (*Cecropin A*: diff = −2.276 [95% CI: −3.078, −1.475], Tukey’s HSD: *P* < 0.001; *Defensin A*: −1.041 [−1.818, −0.264], *P* = 0.015; *Gambicin*: 1.317 [−1.977, −0.657], *P *= 0.002) and at 48 hr pi in comparison with the controls (*Cecropin A*: −2.179 [−2.991, −1.378], *P* < 0.001; *Defensin A*: −1.540 [−2.317, −0.763], *P* = 0.002; *Gambicin*: −1.560 [−2.221, −0.900], *P* = 0.001). However, when comparing 24 hr and 48 hr pi, no significant difference was observed (*Cecropin A*: 0.0969 [−0.705, −0.898], *P* = 0.928; *Defensin A*: −0.500 [−1.276, 0.278,], *P* = 0.200; *Gambicin: −0*.243 [−0.904, 0.417], *P* = 0.532). The expression of *HSP70* in non-infected larvae was similar to that seen in larvae infected with blastospores at 24 hr pi (−0.072 [−0.614, 0.470] *P* = 0.914) but significantly different from infected larvae at 48 hr pi (−1.182[−1.724, −0.640], P = 0.001). A significant difference in expression of *HSP70* between infected larvae at 24 hr pi and 48 hr pi was observed (1.110 [0.568, 1.652], *P* = 0.002).

In *Culex* infected with conidia, the expression of *Cecropin A, Defensin A, Gambicin* and *HSP70* were significantly up-regulated at 12 h pi (*Cecropin A*: diff = −2.516 [95% CI: −3.390, −1.642], Tukey’s HSD: *P* < 0.001; *Defensin A*: −1.203 [−2.286, −0.119], *P* = 0.033; *Gambicin*: −1.975 [−2.718, −1.233], *P* < 0.001; *HSP70: −2*.366 [−3.268, −1.464], *P* < 0.001) and at 24 hr pi in comparison with the controls (*Cecropin A*: −1.853 [−2.727, −0.979], *P* = 0.002; *Defensin A*: −1.380 [−2.463, −0.296], *P* = 0.019; *Gambicin*: −1.866 [−2.608, −1.124], *P* = 0.001; *HSP70: −2*.377 [−3.280, −1.475], *P* < 0.001). The expression levels of *Cecropin A, Defensin A, Gambicin* and *HSP70* at 12 hr and 24 hr were statistically similar (*Cecropin A: −0*.663 [−1.537, 0.211], *P* = 0.127; *Defensin A*: 0.177 [−0.907, 1.260], *P = *0.874; *Gambicin*: −0.109, [−0.852, 0.633], *P* = 0.896; *HSP70*: 0.012 [−0.891, 0.914], *P* = 0.999).

The expression of antimicrobial genes was similar between *Culex* larvae infected with blastospores or conidia at 24 hr pi (*Cecropin A: −0*.240 [−1.161, 0.681], *P* = 0.718; *Defensin A: −0*.162 [−1.189, 0.865], *P* = 0.882; *Gambicin*: −0.549 [−1.281, 0.183], *P* = 0.131, Trans: 0.0417, [−0.727, 0.811], *P* = 0.985). However, the stress management gene *HSP70*, at the same time point, was significantly higher in the larvae infected with conidia than in larvae infected with blastospores (−2.294 [−2.750, −1.838], *P < *0.001).

## Discussion

Larvae of *Cx. quinquefasciatus* are significantly less susceptible to infection by blastospores of *M. brunneum* (ARSEF 4556) than to conidia of the same fungal isolate [3]. This novel study demonstrated some of the possible reasons why *Culex* larvae infected with blastospores take longer to die (LT_50_ = 2.68 days) when compared with those exposed to conidia (LT_50_ = 1.27 days for wet conidia and 1.45 days for dry conidia). We have recently shown that blastospores of this fungus are highly virulent to *A. aegypti* larvae, rapidly penetrating the larval integument with the aid of copious amounts of mucilage [12]. Therefore, we were somewhat surprised to find a totally different infection pattern for *Culex* larvae exposed to *M. brunneum* [3]. The current study was performed with the aim of understanding differences in virulence of blastospores and conidia to *Culex* larvae and also to investigate differences in blastospore interactions with the host when comparing two apparently similar mosquito species. One important factor that will be discussed here is the difference in attachment forces of blastospores to the cuticles of different species of insects. Furthermore, blastospores induce different levels of host immune responses when compared to that seen during conidial infection.

Extracellular proteases produced by *M. brunneum* conidia contribute significantly to the rapid mortality of *Culex* larvae, whilst for blastospores of the same isolate, this is not the case. Differences in virulence between spore types was first observed in *Ae. aegypti* infected with conidia or blastospores as reported by Butt et al. [13] and Alkhaibari et al. [12], respectively. Blastospores attack *Ae. aegypti* larvae through multiple penetration sites with blastospores readily adhering and penetrating the insect cuticle as well as penetrating the gut epithelium following ingestion by the larvae [12]. In contrast, conidia of the same fungus fail to adhere to the larval cuticle but cause death through protease-induced stress following ingestion [13,14]. There were, however, clear differences in the susceptibility of *Cx. quinquefasciatus* and *Ae. aegypti* larvae to conidia in the presence of the protease inhibitor α2-macroglobulin. The inhibitor increased the survival of *Ae. aegypti* larvae exposed to *M. brunneum* conidia significantly more than that of *Cx. quinquefasciatus* larvae, suggesting that the pathogenicity process pathwayto stress induced mortality was not identical in these two mosquito species.

Some studies have shown the correlation between the number of spores adhering to the cuticle and time to host death, with strong adhesion considered an important virulence factor [16,19]. Spore attachment is influenced by hydrophilic and hydrophobic characteristics of the host surface and the fungal cell wall [41]. Holder and Keyhani [22] demonstrated that hydrophilic blastospores of *Beauveria bassiana* bind rapidly to hydrophilic surfaces and bind poorly to hydrophobic surfaces. However, blastospores require a longer incubation time to bind to weakly polar surfaces than to hydrophilic surfaces. In contrast, aerial conidia of *B. bassiana* adhere poorly to weakly polar surfaces and rapidly to both hydrophobic and hydrophilic surfaces but could be readily washed off the latter [22]. Conidia of EPF such as *M. brunneum* and *B. bassian*a, possess hydrophobins in the outer layer of the cell wall whereas these are absent in blastospores [22]. These differences could influence adhesion and consequently impact on EPF efficacy and probably host specificity [22]. The current study shows for the first time that *M. brunneum* blastospores have different degrees of adhesion to the cuticles of different insect host species. AFM data demonstrated that the adhesion forces of the blastospores to *Aedes* larvae were higher than those seen when interacting with *Culex* cuticle. Moreover, the number of attached blastospores on *Culex* larval cuticle, observed using SEM, were much lower in comparison with *Aedes* larvae, which could indicate a weak interaction between the mucilage and *Culex* larval cuticle. The most common sites of blastospore adhesion were on the head and the siphon of *Culex* larvae. Blastospore adhesion at these two sites could be related to the higher levels of sclerotization of these regions when compared to the other regions of the body. The mosquito larval head capsule and siphon are highly sclerotized, but the thorax and abdomen consist of a more flexible cuticle [42]. Furthermore, varying degrees of attachment could also be due to the chemical characteristics of the epicuticle which plays an important role in determining fungal spore adhesion [43]. Hydrophobic lipids and fungistatic compounds in the outermost epicuticular wax layer can play an essential role in attachment and germination of fungal propagules [44–47].

Host cues appeared to influence the expression of pathogenicity related genes in blastospores of *M. brunneum* as demonstrated by the down-regulation of all the target genes to various degrees in the presence of *Culex* larvae, while *Pr1A, Cag8* and *Mos1* are up-regulated in the presence of *Aedes aegypti* larvae [12]. Expression of pathogenicity related genes is also dependent upon the form of the inoculum. Unlike blastospores, expression of pathogenicity related genes in conidia was similar whether exposed to larvae of *Cx. quinquefasciatus, Ae. aegypti* or to a terrestrial host (*T. molitor)* [13]. Variations in gene expression patterns in response to different insect cuticles is well documented for terrestrial insect hosts [48], but the current study shows that this is also true for aquatic insects. The conidia and blastospores appear to respond quickly, often before actually coming into contact with the host, suggesting they are responding to soluble components released by the insect [12,13]. These observations indicate that *M. brunneum* is highly responsive to host cues whether they are at the surface of the cuticle or released into the aquatic environment by the mosquito larvae. These are highly significant findings with importance not only for biological control strategies but also for the evolution of defence mechanisms by insects to entomopathogenic fungi.

Antimicrobial peptides (AMPs), which are produced by the haemocytes and fat body, are considered to be the primary defence elements of the mosquito’s innate immune system. Upregulation of AMPs during infection have been reported [49,50]. Transferrin is a defence molecule which has been shown to be up-regulated in *Cx. quinquefasciatus* during activation of the immune response [51]. Transferrin is an iron-binding protein involved in iron transport, however in insects it also inhibits the growth of bacteria and other invading organisms by sequestering iron [52,53]. The results of the current study showed that the antimicrobial genes; *Cecropin A, Defensin A* and *Gambicin* were significantly up-regulated in both larvae infected with either blastospores or conidia. Although these genes were expressed earlier in larvae infected with conidia (at 12 hr pi), they were still highly susceptible to the conidial infection. Bull et al. [54] reported that one-day old adult bees were highly susceptible to *M. anisopliae* infection, even though they showed a strong immune response. Toll, IMD/JNK and JAK/STAT immune pathways were activated in bees infected with *M. anisopliae* but not healthy control insects [54]. Therefore, a strong immune response may be an indicator of increased insect susceptibility to infection rather than resistance.

Heat shock proteins in insects play a key role in the response to abiotic and biotic stressors. [55] These proteins function as general molecular chaperones that maintain or return proteins to their functional state, minimize aggregation of proteins in their non-native conformation, and target unfolded or aggregated proteins for degradation or removal [56]. *HSP70* has vital housekeeping functions, maintaining homeostasis and protecting cells against thermal and oxidative stress [57] and was up-regulated in *Culex* larvae whether they had been infected with blastospores or conidia suggesting that *HSP70* played a key role in stress management in mosquito larvae. Castillo et al. [58] found that up-regulation of *HSP* genes is evidence of increasing stress in response to pathogen infection. In this study, *HSP70* in *Culex* larvae infected with conidia, was expressed earlier and to a greater extent than in larvae infected with blastospores, which up regulated this gene at 48 hr pi. This indicates that *M. brunneum* conidia induce stress during infection. Butt et al. [13] found that *M. brunneum* conidia attack and kill *Ae. aegypti* larvae by protease-induced stress.

*Culex quinquefasciatus* larvae showed different levels of defence responses to infection by blastospores or conidia of the entomopathogenic fungus *M. brunneum* ARSEF 4556. *Culex tarsalis* larvae showed a strong immune response to *Tolypocladium cylindrosporum* blastospores with hyphae often being enveloped in a thick, dark capsule [11]. Phenoloxidase production, an important humoral immune response to infection, causes melanisation which inhibits pathogens [30]. In this study, *Culex* larvae infected with blastospores produced higher levels of phenoloxidase than that observed during infection with conidia and PO was detected one hour earlier in larvae infected with blastospores when compared to larvae infected with conidia and much earlier than that reported for *Ae. aegypti* larvae [14]. These observations show that not only is the *Culex* immune response different from that of *Aedes* but PO plays a more important role in the former, which is supported by the fact that melanisation at infection sites and of circulating blastospores was only observed in *Culex* larvae.

Some studies reported a possible positive correlation between melanisation, PO activity, and tolerance to parasites and pathogens such as *B. bassiana* and *M. anisopliae* [59,60]. In our electron microscopy studies of the head region, we found that infection of *M. brunneum* blastospores triggers a melanisation response by *Cx. quinquefasciatus* larvae, where melanotic capsules were formed surrounding the blastospores which had invaded the haemocoel and melanisation of the larval cuticle occurred at the contact point between the blastopores and the larval integument. Melanotic capsules surrounding EPF spores have been observed in microscopy studies in several insect species including mosquitoes [30]. Yassine et al. [30] reported that melanisation significantly hinders fungal growth in the mosquito.

Detoxification enzymes such as EST, GST, and SOD can be used by insects to protect themselves from spores and metabolites of EPF by countering oxidative damage and reducing oxidative stress caused by toxicants and accelerating toxin metabolism [40,42]. The activity of most of these enzymes (GST, SOD, and α-EST) were not altered in *Cx. quinquefasciatus* larvae infected with *M. brunneum* conidia, with the exception of ß-esterase at 24 hr pi. However, our study showed that the activities of GST and β-EST were notably higher in larvae infected with blastospores when compared to conidial infections at 24 hr pi. This elevated activity is probably an attempt to inactivate toxic fungal metabolites or the pathogen [39]. Reduced EST and GST activity observed 36 h pi appears to be linked with EPF inhibition of the host defence system [40]. The highest MDA concentration, reflecting lipid peroxidation, was observed on the second day of infection when *Culex* larvae were exposed to blastospores. Increasing MDA concentration indicated an imbalance between pro-oxidant and anti-oxidant activity in the host.

In conclusion, this study is the first to investigate the interactions of two forms of fungal propagule with *Cx. quinquefasciatus* larvae, providing novel insights into their mechanisms of infection and the corresponding defence responses. We have summerised and compared the results of the current study and our previous studies on the pathogenicity of conidia or blastopores against two species of host larvae, *Cx. quinquefasciatus* and *Ae. aegypti* in Table 1. Indeed, analysis of these interactions has provided an explanation for the differential susceptibility of two mosquito species to these two forms of inoculum. *Cx. quinquefasciatus* larvae, unlike *Ae. aegypti* larvae, were less susceptible to infection by blastospores. This was probably due to specific factors related to fungal pathogenicity and specific larval host immune responses. Blastospores kill *Ae. aegypti* larvae by invasion and colonisation of the host via rapid penetration of the integument and gut. However due to the weak attachment of the blastospores to *Culex* larval cuticle, most of the invading blastospores were restricted to infection via the gut. Additionally, *Cx. quinquefasciatus* larvae displayed a stronger and more rapid defence response to blastospores than to conidia. Subtle alterations in host-pathogen interactions can result in significant differences in pathogenicity. These results are important as they will provide guidance to stakeholders deploying EPF in mosquito vector pest control programmes. For example, formulations consisting of a blend of conidia-blastospores may be recommended to treat sites infested with both *Aedes* and *Culex*. These studies have identified key biochemical-molecular events taking place during *M. brunneum* infection of mosquito larvae and established how these influence virulence and specificity. The current study also shows for the first time the ability of a fungus to detect the presence of the host and prepare to launch an attack, whilst the host can also activate defence mechanisms even before physical contact with the pathogen. In the case of predominantly terrestrial fungi such as *Metarhizium*, when attacking aquatic insects, the pathogenicity mechanisms would appear to be in a state of evolution in this arms race.

## Material and methods

### Mosquito source and maintenance

*Culex quinquefasciatus* (Muheza strain) and *Aedes aegypti* (strain AeAe) eggs were obtained from the London School of Hygiene and Tropical Medicine (UK), maintained in tap water in the laboratory at 25°C (±2°C) and a 16L: 8D photoperiod. The larvae were fed on rabbit food (Burgess, Pets at Home Ltd, UK).

*Tenebrio molitor* were kept in ventilated containers being fed organic wheat bran (Holland and Barrett, UK) and kept at 25 (±1°C) and 16 hr: 8 hr photoperiod.

### Fungal production

Conidia and blastospores of *Metarhizium brunneum* ARSEF 4556 were produced in Sabouraud Dextrose Agar (SDA) and Adamek’s media respectively, as described by Alkhaibari et al [12]. Cultivation on SDA medium was used to assess the viability of both conidia and blastospores with only cultures showing ˃95% spore viability used in experiments. An improved Neubauer haemocytometer was used to estimate the spore concentration.

### Susceptibility of *Culex* larvae to Metarhizium blastospores and conidia

Experiments were conducted using 3-4^th^ instar *Cx. quinquefasciatus* larvae. Ten larvae in each of three replicates incubated in 280 mL plastic beakers were exposed to different fungal formulations, 100 mL of conidia and blastospores of *M. brunneum* ARSEF 4556 at a concentration of 10^7^ spores mL^−1^. Conidia were either applied as a dry dust powder over the water surface (dry conidia) or suspended in 0.03% aqueous Tween 80 (wet conidia). Control larvae were exposed to 100 mL of either distilled water or 0.03% aq. Tween 80 in distilled water. Mortality was evaluated daily for 7 days.

### Protease inhibitor assay

Inhibition assays were performed using 24 well-microtitre plates (Nunc, Roskide, Denmark), where one larva was placed in each well (R = 3, N = 72). Each larva was exposed to a 1 mL suspension of blastospores or conidia of *M. brunneum* ARSEF 4556 at a concentration of 10^7^ spores mL^−1^ using the following treatments; (1) blastospores, (2) blastospores incubated with α2-macroglobulin (1 μg ml^−1^, Sigma-Aldrich, St Louise, USA), (3) conidia, (4) conidia incubated with α2-macroglobulin. Controls consisted of either distilled water and distilled water with inhibitor or Tween and Tween with inhibitor. Mortality was recorded daily for four days.

### Light and electron microscope studies

Larvae were exposed to blastospores as described above and examined at 24 and 48 hr post inoculation (pi) to investigate blastospore interactions with the integument and insect gut during the infection process. Infected larvae were examined using light microscopy (LM: Nikon Eclipse 90i microscope), transmission electron microscopy (TEM: JEOL- JEM-1200XII and JEOL 1400 PLUS) and cryo-scanning electron microscopy (SEM: Hitachi S4800). For details on methodology see supplementary files S1, S2 and S3.

### Atomic force microscopy (AFM)

Fungal blastospores were harvested from the growth media and washed twice with distilled water. After washing, spores were freeze-dried and immobilized on a V-shaped tipless silicon nitride cantilever (Bruker Nano Inc.). A JPK nano-wizard II AFM was used to measure the adhesion forces between different larval cuticles (*Aedes, Culex* and *Tenebrio*) and the “blastospore probe” by the vertical deflection of the microscope cantilever. For more details see S4 supplementary file.

### Enzyme activity of *Culex* larvae in response to fungal infection

*Cx. quinquefasciatus* larvae (3 larvae per replicate, n = 9) at different time points post-infection (pi) were homogenized in 100 µL of ice-cold 10 mM sodium phosphate buffer (pH 7.2) containing *N*-Phenylthiourea (PTU 1mgmL^−1^). The homogenates were centrifuged for 5 min, ×10,000 g at 4°C. The supernatants were used immediately to estimate the activity of glutathione-S-transferase (GST), superoxide dismutase (SOD), lipid peroxidase and esterase (EST) according to the method described by Dubovskiy et al [61].

Samples for phenoloxidase (PO) activity estimation were prepared by homogenizing 15 larvae from each of three replicates in 800 μL of phosphate buffer saline (PBS, pH 7.8). Homogenates were centrifuged at 3000 × g for 20 min at 4°C and the supernatants were removed and used immediately in bioassays. Full details are provided in S5.

### Transcript quantification of *Culex* and blastospore-derived genes

Full details of sample preparation, RNA extraction and cDNA preps are provided in supplementary file S6. Briefly, for *Culex* derived genes, three replicates each with ten *Cx. quinquefasciatus* larvae (L3–4) per replicate were exposed to *M. brunneum* ARSEF 4556 blastospores for 24 and 48 h and to conidia for 12 and 24 hr. The time points for larvae infected with conidia were modified since most of the larvae had died at 48 hr pi. Non-infected larvae were used as controls (0 hr). Expression of selected *M. brunneum* genes 24 hr pi was determined in *Culex* larvae inoculated with blastospores and conidia (as described above). For details of the *M. brunneum* pathogenicity/stress and *Cx. quinquefasciatus* immune/stress target genes see supporting information Table S1. Adult *Tenebrio molitor* infected with blastospores and conidia were used as a positive control while non-infected *Culex* larvae were used as a negative control.

All samples were homogenized using a micropestle and total RNA extraction performed using a kit according to manufacturer’s instructions (Qiagen, RNeasy Micro Kit). Purity and concentration of RNA was determined from the ratio A260:280 nm using a Nanophotometer (Implen). Total RNA (1 µg) was reverse transcribed using a QuantiTect Reverse Transcription kit (Qiagen) with gDNA elimination reaction for the experiment to quantify insect-derived transcripts and fungus-derived transcripts, respectively.

Gene transcript levels were determined using a Rotor-Gene 6000 (Corbett Research). Primers were designed to amplify key *Cx. quinquefasciatus* response genes and *M. brunneum* pathology-related genes (SI, Table S1).

## Statistical analysis

Differences in mosquito larval survival when comparing: (1) blastospore and conidial treatments and (2) spores and spores incubated with protease inhibitor were analysed using Kaplan-Meier survival functions by treatment, with pairwise comparison using Log-rank tests. Median lethal times (LT_50_) were estimated by Probit analysis [62]. Differences in enzyme activities were analysed using one-way analysis of variance (ANOVA) with Tukey’s (HSD) post hoc test. A generalized linear model (GLM) was used for statistical analysis of phenoloxidase activity at different time points when compared to non-infected larvae. Prior to analysis, gene expression data was logarithm transformed, conforming to ANOVA assumption of homogeneity of variance [63]. Comparison of adhesion force measurements between samples was analysed using a non-parametric Kruskal-Wallis H test. Pairwise comparisons were performed using Dunn’s post-hoc test for multiple comparisons. All statistical analyses were carried out using SPSS v22.0 [64], R Version 3.3.1 [65] and GraphPad Prism v5.0 (GraphPad Software, USA).
