# Supplementary material for: Highly specific host-pathogen interactions influence Metarhizium brunneum blastospore virulence against Culex quinquefasciatus larvae
Source: Virulence. 2018 Sep 12;9(1):1449–67. doi: 10.1080/21505594.2018.1509665 (PMC6141145; doi:10.1080/21505594.2018.1509665)
Supplement: Supplemental Material [file kvir-09-01-1509665-s001.docx]

**SUPPORTING INFORMATION TEXT**

##### Experimental Procedures

**Text S1 Transmission electron microscopy**

Larvae at various stages of infection and uninfected controls were fixed overnight in a solution consisting of 3% (v/v) glutaraldehyde, 1% acrolein and 0.1% CaCl_2_ in 0.1M PIPES pH 7.4, at 4˚C. The larvae were washed 4 times with 0.2M PIPES pH 7.4 + 0.1% CaCl_2_ at 20 minute intervals then post-fixed in 1% (w/v) osmium tetroxide in 0.1% CaCl_2_ in 0.15 M PIPES buffer pH 7.4 for 1 hr at room temperature. The specimens were washed 3 times with filtered dH_2_O at 20 min intervals before being immersed for 1 hr in 2% (w/v) aqueous uranyl acetate in the dark. The specimens were dehydrated though a graded acetone series (50, 70, 80, 90, 95%) every 20 min before being transferred to 100% dry acetone for 30 mins at RT with five changes during this period. The specimens were then suspended in a graded series of acetone and Spurr’s resin (3:1 for 1 h, 1:1 overnight, 1:3 for 1 h, 100% resin for 6 h on a stirrer, 100% resin overnight). Samples were embedded in 100% Spurr's resin and polymerized at 70 °C for 8 h. Ultrathin (gold) sections were cut using a Reichert ultramicrotome, mounted onto copper grids and examined in a JEOL- JEM-1200XII transmission electron microscope (TEM) at 120 kV (Bath University) and a JEOL 1400 PLUS TEM at 120 kV (UENF).

**Text S2 Cryo-SEM**

*Culex* larvae were mounted on SEM sample holders using cryogenic glue, both horizontally for imaging of the cuticle and vertically for fracturing and cross sectional imaging before being plunged into a nitrogen slush for rapid freezing and then transferred to the cryogenic preparation chamber under vacuum. The specimens were warmed up to -90°C for 10 minutes to remove surface ice then returned to -130°C. Fracturing of the vertically mounted larvae was performed inside the preparation chamber at -130°C using a rotating knife. The specimens were then coated with approximately 5nm of Platinum then transferred to the SEM stage. Imaging took place at -130°C.

**Text S3 Light microscopy of resin embedded sections**

Specimens were prepared as described above and thick sections cut with a Reichert ultramicrotome, stained with general Toluidine Blue cell stain (Microscope Services UK) and examined using a light microscope (Nikon Eclipse 90i). Images were recorded using a digital camera.

**Text S4 Spore probe preparation and atomic force microscopy (AFM)**

Blastospores of *M. brunneum* isolate ARSEF 4556 were harvested from cultures maintained on Adamek's medium [[1](#_ENREF_1)]. Blastospores after freezing-drying were dusted on to a glass slide that had been cleaned with ethanol. Blastopores were immobilized on the end of a V-shaped tip-less silicon nitride cantilever (model MLCT-O10; Bruker AXS, Inc.) following this procedure: (1) A glass bead (Diameter 21.5 μm) was fixed to the AFM cantilever with standard glass glue (Homebase Ltd.) using the AFM instrument; (2) glass glue was then applied to the lower surface of the glass bead using the AFM instrument; (3) freeze-dried blastospores were fixed to the glass bead again using the AFM instrument (Figure S1A & B). To confirm that the blastospores were attached to the glass bead, a light microscope (Ernst Leitz GMBH Wetzlar, Type 020-441.010) was used to visualize the blastospore probe.

The adhesion force measurement between the samples (larval cuticle of *Aedes*, *Culex* and *Tenebrio*) and the blastospore probe was conducted using a JPK nanowizard II AFM (JPK Instruments, Berlin) by the level of vertical deflection of the cantilever. Cantilever deflection was measured as a change in the reflected laser path, resulting in a cantilever deflection -displacement curve [[2](#_ENREF_2)]. To convert the deflection to a quantitative force measurement, the cantilever was calibrated by measuring the spring constant and sensitivity of the cantilever. The spring constant and sensitivity of the cantilever were measured directly by lowering the cantilever onto the surface a Petri dish ([Sterilin™](https://www.thermofisher.com/order/catalog/product/101R20) 50 mm), generating a spring constant of 0.026 N/m and sensitivity of 49.31 nm/V which were used to calculate the force based on Hooke's law *F_force of spring_ = -* (*k_spring constant_*)( *x _displacement_* ). All subsequent AFM force measurements of the samples of interest were carried out in an aqueous environment contained in a liquid cell (Figue S1C). All deflection curves were normalized such that the tip deflection was zero when there was no interaction with the sample surface.


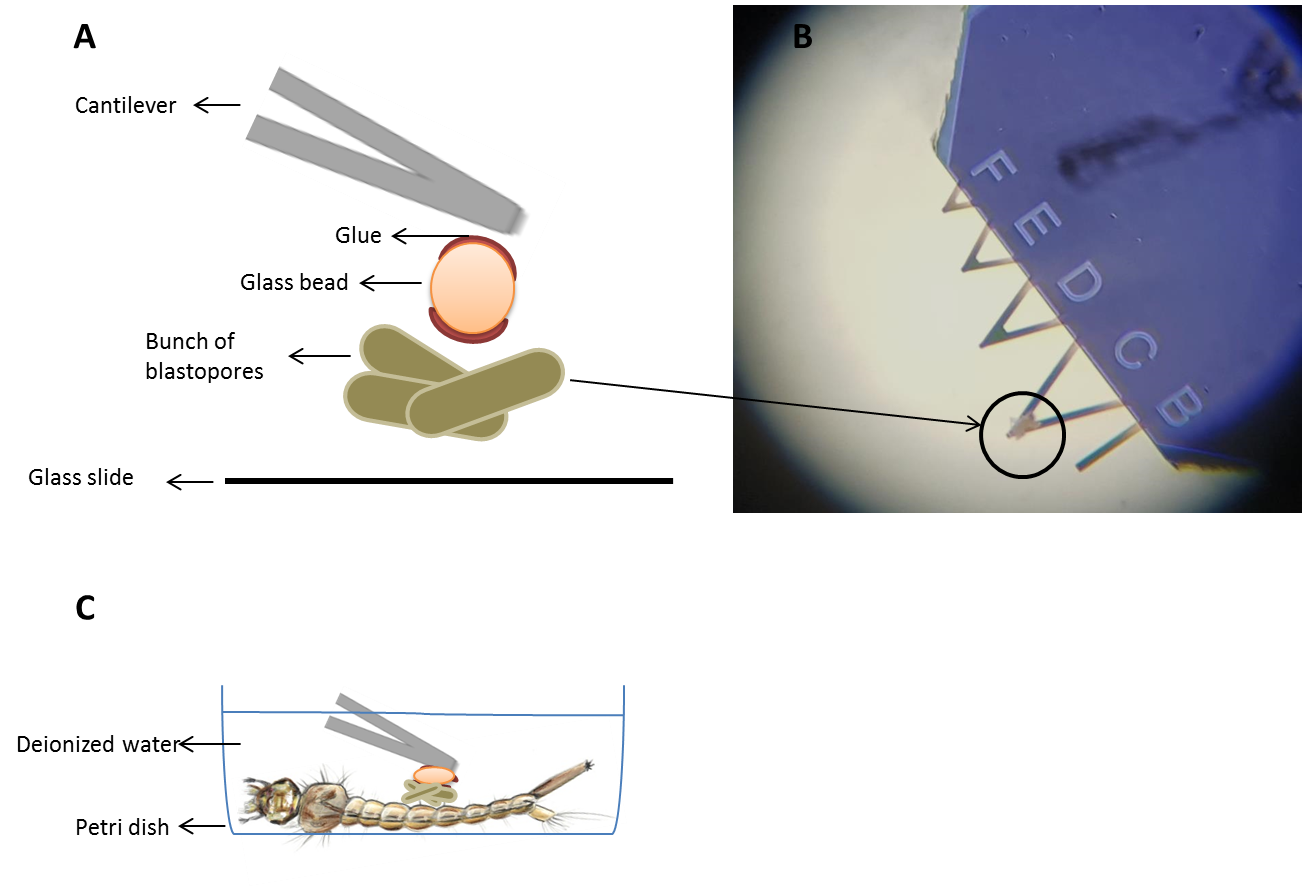


**Figure S1. Principal features of the AFM procedure** A) schematic diagram of the spore probe preparation B) multiple spore probes constructed by immobilization of *Metarhizium brunneum* blastospores on the V-shaped tips of the AFM cantilever. C) Schematic drawing of fluid environment used in all the AFM force measurements.

**Text S5 Insect immune/stress response studies**

***Sample preparation for assays***

Three *Cx. quinquefasciatus* larvae per replicate for each sample were homogenized in 100 µL of ice-cold 100 mM sodium phosphate buffer (pH 7.2) containing *N*-Phenylthiourea (PTU 1mg/mL). The homogenate was centrifuged for 5 min, 10,000 x g (14.000 rpm) at 4 °C. The supernatant was immediately assayed to determine enzyme activities according to the method described by Dubovskiy *et al*. [[3](#_ENREF_3)].

***Enzymes Activities:***

**Glutathione-S-transferase (GST) Activity:** GST activity was determined from alterations in the concentration of 5-(2,4-dinitrophenyl)-glutathione (product of 1-Chloro-2,4-dinitrobenzene (DNCB) and glutathione interaction) catalyzed by GST [[4](#_ENREF_4)]. 2µL of the samples were placed in 96 well microplates. This was followed by addition of 200 µL of 10 mM glutathione reduced prepared in 100 mM sodium phosphate buffer pH 7.2 and 65 mM CDNB dissolved in methanol. The mixture was incubated for 30 min at 28 °С. GST activity is represented as ΔA at 340 nm/min/mg protein.

**Superoxide dismutase (SOD) Activity:** Superoxide dismutase (SOD) activity was determined from the suppression of the reduction rate of NBT by the superoxide anion, generated as a result of xanthine oxidation by xanthine oxidase [[5](#_ENREF_5)]. 2 µL of the samples were mixed with 150 µL of reaction solution (70 μM NBT; 125 μM xanthine; both dissolved in PBS) and 2µL xanthine oxidase solution (10 mg bovine albumin; 100 µl xanthine oxidase; dissolved in 2 mL PBS). The mixture was incubated in the dark at 28 °C for 30 min. SOD activity is presented as at ∆A 560nm /min/mg protein.

**Lipid peroxidation:** The process of lipid peroxidation results in the formation of MDA. This is the last product of the lipid peroxidation reaction sequence [[6](#_ENREF_6), [7](#_ENREF_7)]. A thiobarbituric acid (TBA) assay was used to assess the MDA concentration, with some modifications from that described by Dubovskiy et al [[3](#_ENREF_3)]. 20µL of 20 % trichloroacetic acid was mixed with 40 µL of the sample. The mixture was centrifuged at 10,000 x g for 10 min at 4 °C. 50 µL of supernatant was mixed with 150 µL 0.8% thiobarbituric acid (TBA), and the mixture was incubated at 100 °C for one hour. The MDA-TBA adduct was quantified fluorometrically (Ex/Em = 532/560 nm). The MDA concentration was shown as nmoles of MDA per mg protein using the 1,1,3,3-tetramethoxypropane as a standard.

**General esterase (EST) activity:** The titration level of general esterases was determined according to the method described by Penilla et al. [[8](#_ENREF_8)] with some modifications. Briefly, α **/** β naphyl-acetate substrate was used. 200 µL of α **/** β naphyl-acetate solution [120 µL of 30 mM α- or β-naphyl-acetate dissolved in 12 mL of PBS] was added to 2 µL of the sample and incubated in the dark at room temperature for 30 min. After incubation, a mixture of 50 μL of 0.023g fast blue salt dissolved in 2.25 mL distilled water plus 5.25 mL 5% sodium lauryl sulphate (SDS) dissolved in phosphate buffer (pH 7.0) was added. The enzyme activity was shown as ΔA at 590 nm/min/mg protein.

**Phenoloxidase (PO) Activity:** Phenoloxidase activity was detected in larval homogenates [[9](#_ENREF_9), [10](#_ENREF_10)] during a 24 h period post *Metarhizium* infection (Time zero, 30 min, 40 min, 50 min, 1 h, 2 h, 3 h, 4 h, 5 h, 6 h, 12h, and 24 h). Larvae were removed at each time point, frozen under liquid nitrogen and stored at -80°C until required. 15 larvae per replicate (infected or controls) were homogenised in 1.5 Eppendorf tubes with 300 μL of phosphate buffer saline (PBS, pH 7.8), then 500 µL of PBS buffer was added to the homogenate. Homogenates were centrifuged at 3000 x g for 20 min at 4 °C and the supernatants were removed and used immediately for enzyme assays.

3, 4-dihydroxy-L-phenyl-alanine (L-DOPA) was used as a substrate to determine PO activity. PO activity is expressed as a change in absorbance/min/mg protein [[11](#_ENREF_11)]. 20 μL of larval homogenate was added to 180 μL of L-DOPA (4mgmL^-1^) in a 96-well plate and incubated at room temperature for 30min. End point absorbance was measured at 490nm.

The concentration of protein in the homogenates was determined by the Bradford method [[12](#_ENREF_12)] and bovine serum albumin (BSA) was used to construct the calibration curve. All assays were performed using a Biotek Synergy H1 plate reader.

**Text S6 Gene Expression studies**

**Sample preparation:** Blastospores were cultured as previously stated and collected by centrifugation (MICRO 22) of fungal liquid cultures at x 18,000 g for 5 min. Ten *Cx. quinquefasciatus* larvae from each of three replicates were exposed to a 100 mL blastospore or conidial suspension at a concentration of 10^7^ spores mL^-1^ in 250 mL circular plastic containers and incubated for 24 h and 48 h for blastospores and 12 and 24 h for conidia at room temperature (25±2 °C). Controls not exposed to fungus were also incubated at the same time points. Ten larvae per replicate were frozen under liquid nitrogen and stored at -80°C until required.

For fungal gene expression determinations, *Culex* larvae were exposed to *Metarhizium* blastospores and conidia as described above for 24 h. Other treatments included: blastospores and conidia incubated for same time period but in the absence of larvae, and a terrestrial host (*Tenebrio molitor*) infected with blastospores and conidia was used as positive control. Adult *T. molitor* were submerged in 10 mL of 10^7^ spores mL^-1^ for 20 sec and incubated on moist filter paper for 24 h at room temperature before freezing under liquid nitrogen. All the samples from three replicates were frozen in liquid nitrogen and stored at -80°C until required.

**RNA isolation and cDNA synthesis:** Samples were homogenized with a micropestle and RNA was extracted using an RNeasy Micro kit (Qiagen) according to manufacturer instructions. Purity and concentration of RNA was determined by using a Nanodrop 2000 (Thermoscientific) based on the ration of 260/280 nm. RNA was stored at -80°C until required. Total RNA (1μg) was transcribed into cDNA using a QuantiTect Reverse Transcription kit (Qiagen) with a gDNA elimination reaction.

**Real time qPCR:** The relative quantity cDNA was determined by using a qPCR method as previously described [[13](#_ENREF_13)]. PCR amplification of all genes was performed in a total volume 10 µL containing the following: 1μM of each primer, 2μL of diluted cDNA, 5μL SYBR (Invitrogen Green Fastmix, Quanta) and 1μL UltraPure™ DNase/RNase-Free Distilled Water™ (Gibco). The housekeeping genes for *Culex* derived genes were 18S ribosomal and 60S ribosomal protein L8, while fungus derived genes were 18s (ribosomal RNA), and TEF (translation elongation factor). The genes investigated in this study were Gambicin, Transferrin, Defensin A, Cecropin A, and heat shock protein 70 (*HSP* *70*) for *Culex* and *Mad1*, *Mad 2*, *Pr1 A*, *Pr2*, *Cag8, nrr1, Mos1,* *HSP* *30*, *HSP* *70* and *HSP* *90* for *M. brunneum* blastospores (Table S1). The PCR amplification conditions were 95°C for 5 minutes, 42 cycles at 95°C for 5 sec, primer annealing (64-59°C) for 10 sec, 72°C for 20 sec. Transcript levels were determined by using the Rotor-Gene 6000 system. A high resolution melt curve (HRM) analysis was included for all reactions to ensure no spurious products were present. Non-template water was used as a negative control in each run to ensure no contamination in the PCR reagents. Two-fold dilution series of pooled cDNA samples (at a concentration of 32, 16, 8, 4, 2, and 1 ng/mL) was used for each run to provide a standard curve for each primer. The efficiency of RT-PCR was calculated by generating a standard curve plotting ct values against the log template amount, and then using the slope of the standard curve. The following formula was used to calculated the efficiency: PCR efficiency (%) = 10^(-1/5)^ -1) × 100. Samples with Ct (cycle threshold) values higher than non-template control were considered as zero. The samples were run in triplicate and replicates were run in duplicate.

|  | **Function** | **Locus** | **Accession number** | **Primer name** | **Forward primer** | **Reverse Primer** | **Reference** |
| --- | --- | --- | --- | --- | --- | --- | --- |
| *Cx. quinquefasciatus* genes | Antimicrobial peptides | Gambicin | AY388563 | Cu_Gamb | CTGTGACGACTGCAGGAGAC | AATCCTCGCTGAGCTCTCGT | [[14](#_ENREF_14)] |
|  | Immune system-associated | Transferrin | DQ400917.1 | Cu-Trans | AAGTACTCTCCGAACGACGA | CCGAGTACTTGTCCGGGTAG | [[14](#_ENREF_14)] |
|  | Antimicrobial peptides | Defensin A | XM_001842893.1 | Cu_DefA | CGTACCAAGCCGCCGCAGAG | GCCGCGCAGGCACTATCGTT | [[15](#_ENREF_15)] |
|  | Antimicrobial peptides | Cecropin A | XM_001861705.1 | Cu_CecA | GGTCTGAAGAAGTTTGGCAAGA | CGCCTTGAATCCAGTAACGA | Our lab |
|  | Stress | heat shock protein 70 B2 | XM_001861401.1 | Cu_HSP 70 | CCGAGAAGGACGAGTTTGAG | ATCAACTTCCTCAACGGTGG | [[16](#_ENREF_16)] |
|  | Housekeeping gene | 18S Ribosomal | AY988447.1 | Cu-18S | CGCGGTAATTCCAGCTCCACTA | GCATCAAGCGCCACCATATAGG | [[17](#_ENREF_17)] |
|  | Housekeeping gene | 60S ribosomal protein L8 | XP_001841927 | Cu-Rb60sL8 | AGTCGTGAAGCACATCATCCACG | GCCTTACCGATGTGCTGATGGTT | [[18](#_ENREF_18)] |
| *M. brunneum* genes | Adhesion | Adhesion | MBR_08250 | *Mad 1* | CTCCTCACATCACCCAGGTT | GGGAGTAGGCATGACGATGT | [[13](#_ENREF_13)] |
|  | Adhesion | Adhesion | DQ338438.1 | *Mad 2* | CTATGTCCACCTTGCGACT | AGCAGCTGATGAGGGTCT | [[13](#_ENREF_13)] |
|  | Protease | Protease | MBR_01491 | *Pr1 A* | GATTGGTGGCAGCACTAAC | TCCTGGATCTTCTTGCAAAG | [[19](#_ENREF_19)] |
|  | Protease | Protease | MBR_06579 | *Pr2* | TACGCCACATTGCCAGAG | GCATGTCGCACGATCAAC | [[13](#_ENREF_13)] |
|  | Stress | Stress | DQ393581.1 | *HSP 30* | GGTCCAACGCATCACACT | CTTCTTCTCCTCGGGCTCA | Our lab |
|  | Stress | Stress | DQ393579.1 | *HSP 70* | CTGTCAACAATGCCGTCATCA | ATATCATCCTTGTCCTTGTCCTC | Our lab |
|  | Stress | Stress | DQ393580.1 | *HSP 90* | GTGGTACCTTCAGCATCACC | CTTCTGGACGTGGAGGTAGA | Our lab |
|  | Housekeeping gene | 18S ribosomal | DQ288247.1 | 18s | CGAAAGTCGCAATGGCTCA | CCGAAGTCGGGATTTTTAGC | [[13](#_ENREF_13)] |
|  | Housekeeping gene | translation elongation factor | MBR_08275 | TEF | CGAGCGTGAGCGTGGTA | CAGCCTCGAACTCACCAG | [[13](#_ENREF_13)] |

**Table S1** Target *M. brunneum* and *Culex quinquefasciatus* genes and primers for quantitative PCR

**References**

1. Adamek L. Submerse cultivation of the fungus *Metarhizium anisopliae* (Metsch.). Folia Microbiol (Prah) 1963; 10:255-257

2. Greenfield BP, Lord AM, Dudley E, et al. Conidia of the insect pathogenic fungus, *Metarhizium anisopliae*, fail to adhere to mosquito larval cuticle. Royal Soc Open Sci 2014; 1:140193

3. Dubovskiy I, Martemyanov V, Vorontsova Y, et al. Effect of bacterial infection on antioxidant activity and lipid peroxidation in the midgut of *Galleria mellonella* L. larvae (Lepidoptera, Pyralidae). Comp Biochem Physiol C Toxicol Pharmacol 2008; 148:1-5

4. Habig WH, Pabst MJ, Fleischner G, et al. The identity of glutathione S-transferase B with ligandin, a major binding protein of liver. PNAS 1974; 71:3879-3882.

5. McCord JM, Fridovich I. Superoxide dismutase an enzymic function for erythrocuprein (hemocuprein). J Biol Chem 1969; 244:6049-6055

6. Rael LT, Thomas GW, Craun ML, et al. Lipid peroxidation and the thiobarbituric acid assay: standardization of the assay when using saturated and unsaturated fatty acids. BMB Reports 2004; 37:749-752

7. Evans PJ, Gallesi D, Mathieu C, et al. Oxidative stress occurs during soybean nodule senescence. Planta 1999; 208:73-79

8. Penilla PR, Rodriguez AD, Hemingway J, et al. Resistance management strategies in malaria vector mosquito control. Baseline data for a large‐scale field trial against *Anopheles albimanus* in Mexico. Med Vet Entomol 1998; 12:217-233

9. Ahmed AM. Immune and cellular impacts in the autogenous *Aedes caspius* larvae after experimentally-induced stress: Effects of *Bacillus thuringiensis* infection. J Basic Appl Zool 2013; 66:1-11

10. Li JS, Kim SR, Christensen BM, et al. Purification and primary structural characterization of prophenoloxidases from *Aedes aegypti l*arvae. Insect Biochem Mol Biol 2005; 35:1269-1283

11. Dubovskiy IM, Whitten MM, Yaroslavtseva ON, et al. Can insects develop resistance to insect pathogenic fungi? PLoS One 2013; 8:e60248

12. Bradford MM. A rapid and sensitive method for the quantitation of microgram quantities of protein utilizing the principle of protein-dye binding. Anal Biochem 1976; 72:248-254

13. Butt TM, Greenfield BP, Greig C, et al. *Metarhizium anisopliae* pathogenesis of mosquito larvae: a verdict of accidental death. PLoS One 2013; 8:e81686.

14. Vézilier J, Nicot A, Lorgeril J, et al. The impact of insecticide resistance on *Culex pipiens* immunity. Evol Appl 2013; 6:497-509

15. Paradkar PN, Trinidad L, Voysey R, et al. Secreted Vago restricts West Nile virus infection in *Culex* mosquito cells by activating the Jak-STAT pathway. PNAS. 2012; 109:18915-18920.

16. Zhao L, Becnel JJ, Clark GG, et al. Identification and expression profile of multiple genes in response to magnesium exposure in *Culex quinquefasciatus* larvae. J Med Entomol 2010; 47:1053-1061.

17. Liu N, Li T, Reid WR, et al. Multiple cytochrome P450 genes: their constitutive overexpression and permethrin induction in insecticide resistant mosquitoes, *Culex quinquefasciatus.* PLoS One 2011; 6:e23403.

18. Pelletier J, Leal WS. Genome analysis and expression patterns of odorant-binding proteins from the Southern House mosquito *Culex pipiens quinquefasciatus*. PloS One 2009; 4 :e6237.

19. Fang W, Bidochka MJ. Expression of genes involved in germination, conidiogenesis and pathogenesis in *Metarhizium anisopliae* using quantitative real-time RT-PCR. Mycol Res 2006; 110:1165-1171.
